# Combined Machine Learning and GRID-Independent Molecular Descriptor (GRIND) Models to Probe the Activity Profiles of 5-Lipoxygenase Activating Protein Inhibitors

**DOI:** 10.3389/fphar.2022.825741

**Published:** 2022-03-01

**Authors:** Hafiza Aliza Khan, Ishrat Jabeen

**Affiliations:** Research Centre for Modelling and Simulation (RCMS), NUST Interdisciplinary Cluster for Higher Education (NICHE), National University of Sciences and Technology (NUST), Islamabad, Pakistan

**Keywords:** 5-lipoxygenase activating protein (FLAP) inhibitors, machine learning, molecular docking, grind, leukotrienes (LTs)

## Abstract

Leukotrienes (LTs) are pro-inflammatory lipid mediators derived from arachidonic acid (AA), and their high production has been reported in multiple allergic, autoimmune, and cardiovascular disorders. The biological synthesis of leukotrienes is instigated by transfer of AA to 5-lipoxygenase (5-LO) *via* the 5-lipoxygenase-activating protein (FLAP). Suppression of FLAP can inhibit LT production at the earliest level, providing relief to patients requiring anti-leukotriene therapy. Over the last 3 decades, several FLAP modulators have been synthesized and pharmacologically tested, but none of them could be able to reach the market. Therefore, it is highly desirable to unveil the structural requirement of FLAP modulators. Here, in this study, supervised machine learning techniques and molecular modeling strategies are adapted to vaticinate the important 2D and 3D anti-inflammatory properties of structurally diverse FLAP inhibitors, respectively. For this purpose, multiple machine learning classification models have been developed to reveal the most relevant 2D features. Furthermore, to probe the 3D molecular basis of interaction of diverse anti-inflammatory compounds with FLAP, molecular docking studies were executed. By using the most probable binding poses from docking studies, the GRIND model was developed, which indicated the positive contribution of four hydrophobic, two hydrogen bond acceptor, and two shape-based features at certain distances from each other towards the inhibitory potency of FLAP modulators. Collectively, this study sheds light on important two-dimensional and three-dimensional structural requirements of FLAP modulators that can potentially guide the development of more potent chemotypes for the treatment of inflammatory disorders.

## 1 Introduction

The 5-LO pathway is responsible for the biological synthesis of leukotrienes (LTs) using arachidonic acid (AA) predominately by inflammatory cells like polymorphonuclear leukocytes, activated macrophages, and mast cells upon arrival of immunologic and non-immunologic stimuli (Hedi and Norbert 2004). Activation of leukocytes results in translocation of cytosolic protein phospholipase A2 (PLA2) to membrane where it selectively hydrolyzes the sn-2 acyl bond of membrane phospholipids to release AA and lysophosphatidic acid. An integral membrane protein called FLAP (5-lipoygenase activating protein) uptakes the AA and efficiently transfers it to the active site of 5-lipoxygenase (5-LO) enzyme, which catalyzes a series of reactions at a single active site (Peters-Golden 1998; Peters-Golden and Brock 2003). In the first step, in a calcium- and ATP-dependent reaction, AA is converted to a 5-lipoxygenase-specific hydroperoxide intermediate (5-HPETE), while in the second step, 5-LO performs synthase reaction for conversion of 5-HPETE to the epoxide intermediate, leukotriene A4 (LTA4) (Woods et al., 1995; Smyrniotis et al., 2014). LTA4 acts as a common precursor for biosynthesis of chemoattractant leukotriene B4 (LTB4) by a zinc-bound LTA4 hydrolase (LTA4H) and bronchoconstrictive cysteinyl leukotrienes (CysLTs or LTC4) with the help of LTC4 synthase (LTC4S) (Jakschik and Kuo 1983; Haeggström 2000). Both LTB4 and CysLTs are physiologically active final products of the 5-LO pathway and are exported out of the cell through specific transport proteins while extracellular peptidases metabolize LTC4 to LTD4, which is converted into LTE4 depending on type of inflammatory signal and cell demand (Jedlitschky and Keppler 2002). After export, LTs bind with respective G-protein-coupled receptors, e.g., LTB4 binds with BLT1 and BLT2, whereas CysLTs activate CysLT1 and CYsLT2 receptors to incite further proinflammatory signaling cascades (Ghosh et al., 2016).

Since high levels of LTs have been reported in the pathophysiology of a wide range of inflammatory, cardiovascular, and autoimmune disorders, FLAP has become the focus of immense research because LT production can be stopped at the earliest level (Folco et al., 2000; Liu and Yokomizo 2015). Over the course of the last 3 decades, several FLAP modulators have been proposed including first generation of derivatives of indoles and quinolines for asthma treatment (Evans et al., 1991; Frenette et al., 1999). These inhibitors such as MK-886, MK-591, and BAY-X-1005 demonstrated efficiency in clinical trials in patients with inflammatory diseases in the mid-1990s but were not brought to market due to poor pharmacokinetics (Friedman et al., 1993; Diamant et al., 1995; Dahlén et al., 1997). Revelation of SAR data along with crystal structure expedites the drug discovery quest against FLAP, leading to the second generation consisting of derivatives of diarylalkanes, biaryl amino-heteroarenes, and benzimidazoles, proposed with renewed interest for treatment of cardiovascular diseases (Lemurell et al., 2015; Macdonald et al., 2008; N.; Stock et al., 2010). Moreover, several inhibitors proved to be promising readouts for preclinical and clinical studies such as AM103, AM803, BI665915, AZD5718, and AZD6642 and have been shown to ameliorate inflammation-related diseases (Bain et al., 2010; Lorrain et al., 2010; Antoniu, 2014; Ericsson et al., 2020). However, despite several practices, not a single inhibitor has won the race to the market as a drug to date. Therefore, development of more potent chemical entities against FLAP is highly desirable to provide relief to patients suffering from inflammatory disorders.

Mostly FLAP modulators were synthesized and pharmacologically tested and optimized through SAR (structure–activity relationship) studies. Some candidates were also identified by virtual screening from a ligand-based pharmacophore built upon smaller datasets (Banoglu et al., 2012; Temml et al., 2017; Olgac et al., 2020). Here, in this study, advanced machine learning (ML) techniques along with classical modeling strategies are adapted to shed light on important 2D and 3D anti-inflammatory properties of a diverse set of inhibitors targeting FLAP. For this reason, ML models based on most relevant 2D descriptors or features have been constructed. Further molecular docking was performed to establish a binding hypothesis of each class of inhibitors within the FLAP binding cavity followed by common scaffold clustering to obtain the most probable 3D binding solutions. The most probable 3D binding poses were utilized for GRID-independent molecular descriptor analysis (GRIND) to probe the important 3D binding features and associated mutual distances in active FLAP modulators.

## 2 Materials and Methods

### 2.1 Machine Learning Modeling

#### 2.1.1 Dataset Preparation

All compounds having activity values in IC_50_ against FLAP were retrieved from the ChEMBL database under target ID ChEMBL4550 followed by removal of compounds with similar canonical smiles resulting in a dataset of 658 compounds. The IC_50_ of the finalized 658 compounds ranged from 0.3 to 22,500 nM. Furthermore, the highly active and least active compounds were distinguished by the application of activity threshold, i.e., compounds having IC_50_ < 10 nM were categorized as highly active while compounds having IC_50_ > 70 nM were categorized as least active considering that FLAP inhibitors that have entered clinical trials usually possess values < 10 nM (Gür, Çalışkan, and Banoglu 2018). Compounds with IC_50_ values in between >10 nM and <70 nM were labeled as intermediates and were removed. For ML classification model development, highly active compounds were labeled as one, while least active ones were labeled as 0. The final dataset was composed of 503 (253 highly actives and 250 least actives) compounds and was randomly divided into a training set (402 compounds: 201 highly actives and 201 least actives) and a test set (101 compounds: 52 highly actives and 49 least actives) by a ratio of 80% and 20% respectively using *train_test_split* function (*random_state* = 42) of model_selection library from the scikit-learn Python package (Pedregosa et al., 2012). Additionally, it was ensured that the ratio of the highly active to weakly active inhibitors remained equal in the training and test set.

#### 2.1.2 Computation of 2D Chemical Descriptors

Initially, 4,179 2D descriptors were calculated using alvaDesc tool version 2.0.8 (Mauri 2020). The descriptors can be divided into 21 categories named constitutional indices, ring descriptors, topological indices, walk and path counts, connectivity indices, information indices, 2D-matrix based descriptors, 2D autocorrelations, burden eigenvalues, P_VSA like descriptors, ETA indices, edge adjacency indices, fractional group counts, atom-centered fragments, atom-type-estate indices, pharmacophore descriptors, 2D atom pairs, charge descriptors, molecular properties, drug-like indices, MDE descriptors, and chirality descriptors. Descriptors with null values and variance near zero were removed. For the remaining 2,352 descriptors, Pearson autocorrelation coefficient was calculated and autocorrelated descriptors along with low dependency (correlation) on the target variable (inhibitory potency, IC_50_) were discarded, resulting in a set of 442 descriptors. The final 442 features were further applied to train the ML models.

#### 2.1.3 Machine Learning Modeling

For this study, six supervised ML classification models named support vector machine (SVM), random forest (RF), multilayer perceptron (MLP), decision tree (DT), logistic regression (LR), and gradient boost decision tree (GBDT) were developed. SVM, RF, MLP, DT, and LR were generated using the scikit-learn Python package whereas the GBDT was built by the XGBoost Python package (Cortes, Vapnik, and Saitta 1995; Liaw and Wiener 2002; Haykin, 2009; Quinlan 1986; T.; Chen and Guestrin 2016; McCullagh and Nelder 1989). To select the most relevant features from the set of 442 descriptors, the RFECV (Recursive Feature Elimination and Cross-Validation Selection) algorithm of scikit-learn was used (Guyon et al., 2002). Recursive feature elimination (RFE) is a wrapper-type feature selection that works by eliminating n features from a model by fitting the model multiple times and, at each step, removing the weakest features, determined by either the *coef_* (SVM and LR) or *feature_importances_* (RF, DT, and XGBoost) attribute of the fitted model (Guyon et al., 2002). Since there is no attribute available to estimate feature importance in MLP, XGBoost was used as the base estimator. The cross-validation (*cv*) parameter of RFECV was set at fivefold and was done by using the RepeatedStratifiedKFold method of the model_selection library from scikit-learn (Zeng and Martinez 2010). The GirdSearchCV library in scikit-learn was used to tune hyperparameters of the estimators based on a 10-fold cross-validation Matthews Correlation Coefficient (MCC). This process was repeated ten times. Moreover, to assemble data transformer (RFECV) and hyperparameter tuner (GirdSearchCV) with simultaneous cross-validation while setting different parameters, the pipeline module of scikit-learn was used.

An SVM constructs a maximum marginal hyperplane with the help of a kernel function to map the non-linear problem in multidimensional space for data separation. The performance of the SVM model is controlled by parameters such as kernel, capacity parameter (*C*), and gamma. Kernel represents sample distribution in the mapping space, C controls the trade-off between smooth decision boundary, and gamma controls the extent of curvature in decision boundary (Nekoei, Mohammadhosseini, and Pourbasheer 2015; Pourbasheer et al., 2017). For this project, linear kernel was utilized while all parameters were set at their default values except for tuning of penalty parameter (*C*) (Chang and Lin, 2021). MLP is a feedforward artificial neural network and is trained using back propagation algorithm. It has an activation function that forms a linear combination according to weights of inputs to decide the output. The MLP model was controlled by tuning the following parameters: the number of neurons (*hidden_layer_sizes*) and activation function (*activation*), while the rest of the parameters were set at their default values (Glorot and Bengio, 2021). An LR model predicts a dependent data variable by analyzing the relationship through logic functions between one or more existing independent variables. It was controlled by tuning the following parameters: the way of regularization (*penalty*), strength of regularization (*C*), tolerance for stopping criteria (*tol*), and algorithm of optimization (*solver*), whereas other parameters not mentioned were set at their default values (Fan et al., 2008). A DT classifies data by splitting them into source nodes and then multiple child nodes using statistical probability. The DT model was optimized by tuning the following parameters: quality of split (*criterion*), split at each node (*splitter*), and number of features for the best split (*max_features*). The remaining parameters were set as their default values (Brieman and Olshen 2012). An RF builds multiple decision trees and merges them together to get an accurate and stable prediction. The RF model was controlled by tuning the following parameters: number of trees (*n_estimators*), quality of a split (*criterion*), features for the best split (*max_features*), and the minimum number of samples required for splitting (*min_samples_split*); the other parameters not mentioned were set at their default values (Breiman 2001). XGBoost is an ensemble tree method that applies the principle of boosting weak learners using the gradient descent architecture. For this project, gradient boost tree (GBDT) has been implemented, which uses decision trees as weak classifiers. The XGBoost model was controlled by tuning the following parameters: the maximum depth of a tree (*max_depth*), the number of the tree (n_estimators), minimum loss reduction required for partition on a node (*gamma*), minimum sum of instance weight needed to generate a child node (*min_child_weight*)*,* strength of L1 regularization (*reg_alpha*), and learning rate (*learning_rate*). The other parameters not mentioned were set at their default values (T. Chen and Guestrin 2016).

The repeated stratified 5-fold cross-validation was used on the training set to select and evaluate the robustness of models, and the test set was used to evaluate the performance of models. Evaluation parameters include classification accuracy (ACC), true positive rate or sensitivity (SE), true negative rate or specificity (SP), and Matthews correlation coefficient (MCC) as mentioned in (Eqs 1–4) below:

$$True\;Positive\;Rate\;\left(Sensitivity\right)=\frac{TP}{TP+FN}$$ (1)

$$True\;Negative\;Rate\;\left(Specificity\right)=\;\frac{TN}{TN+FN}$$ (2)

$$Classification\;Accuracy\;\left(ACC\right)=\frac{TP+TN}{TP+TN+FP+FN}\;$$ (3)

$$Matthews\;Correlation\;Coefficient\;\;\left(MCC\right)=\frac{\left(TP\times TN-FP\times FN\right)}{\sqrt{\left(TP+FP\right)\left(TP+FN\right)\left(TN+FP\right)\left(TN+FN\right)}}$$ (4)

### 2.2 Molecular Modeling

#### 2.2.1 Calculation of Lipophilic Efficiency (LipE) and cLogP

To estimate the druglikeness of the initially finalized 658 FLAP inhibitors (section 2.1.1), LogP was calculated by using Bio-Loom software (BioByte - Bio-Loom, 2021) followed by computation of LipE with the following equation:

$$LipE=pIC_{50}-c\log P$$ (5)

Briefly, lipophilicity or cLogP strongly impacts membrane passive permeability, which is required for oral absorption and access of the drug to intracellular compartments and tissue penetration (Arnott and Planey 2012). Lipophilic efficiency (LipE) is defined as normalization of the pIC_50_ with respect to cLogP of the compound. Previously, Leeson et al. proposed that an ideal drug candidate should have a LipE value greater than five, which is obtained in case of high potency and low lipophilicity (Leeson and Springthorpe 2007). For the application of molecular modeling techniques (Docking and 3D QSAR GRIND), LipE and cLogP filter were used; i.e., compounds having LipE greater than one and cLogP greater than two were selected. The new dataset of compounds having LipE value greater than one and cLogP greater than two was divided into a training set (80%) and a test set (20%) by using the *train_test_split* function (*random_state* = 42) of the model_selection library from the scikit-learn Python package. Both training and test datasets were further employed in molecular modeling studies (docking studies and GRIND modeling).

#### 2.2.1 Molecular Docking and Pose Analysis

To explore the binding interactions of structurally diverse FLAP inhibitors, and to obtain the most probable 3D binding conformations of ligands for GRIND analysis, inhibitors having LipE value greater than one and cLogP greater than two were docked into the binding pocket of the FLAP structure retrieved from the Protein Data Bank (PDB ID: 2Q7M) (Ferguson et al., 2007). Protein structure was prepared by energy minimization through the Amber99 force field of MOE (A. A. Chen and Pappu 2007). The energy-minimized structure was imported into GOLD software (version 5.6.1) (Jones et al., 1997) followed by determination of x, y, z coordinates around the single-solvent accessible point present in the center of the active site. The binding site area was kept at 12 Å radius, which included all important amino acid residues reported by previous studies. A total of 100 conformations for each ligand were generated, and GOLD fitness scoring function was used to rank each pose of ligands with subsequent energy minimization of ligand–protein docking complexes using LigX implemented in software MOE. Gold score fitness scoring function was calculated as:

$$Fitness=S_{{\left(hb\right)}_{ext\;\;}}+1.3750*S_{{\left(vdw\right)}_{ext\;\;}}+S_{{\left(hb\right)}_{\mathrm{int}\;}}+1.0000*\;S_{\left(\mathrm{int}\right)}$$ (6)

Based on structural similarity, common scaffold clustering (CSC) as proposed by Jabeen et al. (2012) was conducted to reduce the conformational space. For this purpose, RMSD matrix was generated through agglomerative hierarchical cluster analysis, and clusters with maximum docked ligands were selected for ligand–protein interaction profiling. Common interactions between each class were sorted out and binding hypothesis was generated for each class with respect to interaction pattern and position in binding pocket. Conformations from selected clusters were further utilized in GRIND analysis as training set.

#### 2.2.3 Grid Independent Molecular Descriptors Analysis

Selected 3D molecular confirmations of ligands obtained from clusters containing maximum docked ligands along with their inhibitory potencies (pIC_50_) were imported in Pentacle software version 1.06 to construct the GRIND model (Pastor et al., 2000). Calculation of molecular interaction fields (MIFs) was done by use of different probes, namely, N1, O, DRY, and TIP, where N1 (amide N) represents a hydrogen bond donor, O (sp^2^ carbonyl O) denotes a hydrogen bond acceptor, DRY indicates a hydrophobic region, and TIP stands for steric hotspots within the virtual receptor site. A GRID was used to iteratively place these probes to calculate the total energy by addition of Lennard-Jones potential energy (Elj), hydrogen bond energies (Ehb), and the electrostatic energy (Eel), whereas with the help of the following equation, total interaction energy at each node was calculated:

E_xyz=∑E_hb+∑E_lj+∑E_el (7)

AMANDA algorithm was used to extract the most relevant and significant MIFs along with evaluation of structural characteristics of the dataset explained by GRIND descriptors (Durán, Martínez, and Pastor 2008). The default GRID space of 0.5 and the energy cutoff values, which are –4.2, –2.6, –0.5, and –0.74 for N1, O, DRY, and TIP, respectively, were used for discretization of MIFs, while nodes that did not meet the energy cutoff were discarded. The next encoding step involves CLACC algorithm that aided in selection of consistent nodes by adjustment of compounds according to their moment of inertia. The values obtained from encoding consist of a consistent set of variables whose values were directly represented in the form of correlogram plots. The final GRIND model with PLS (partial least square) analysis using LOO (leave one-out) method with statistically significant *R* ^2^, *q* ^2^, and standard error values (SDEP) was built on the training set followed by evaluation with the test set (section 2.2.1). Additionally, r^2^m metrics (r^2^m, Delta r^2^m) was also generated for validation purposes according to the previously published studies (Roy et al., 2013; Gajo et al., 2016).

## 3 Results

### 3.1 Machine Learning Models

Six ML models were developed by different algorithms (SVM, LR, MLP, DT, RF, and GBDT) using two-dimensional structural features of FLAP inhibitors. The performance of these models on 5-fold repeated stratified cross-validation is explained in Table 1. The cross-validation accuracy of the training set ranged between 0.90 and 0.75, and the MCC ranged from 0.81 to 0.50. The prediction accuracy and MCC values of the test set ranged from 0.90 to 0.70 and 0.80 to 0.40, respectively. MCC is often used as a measure of quality of binary classification models. Two models (XGBoost and RF) exhibited an MCC value of >0.7 on training and test sets, which means these two algorithms have a relatively good ability to predict whether a compound was a highly active or a least active FLAP modulator. In terms of the best model, XGBoost outperformed all and the accuracy and MCC values were observed as 0.90 and 0.81, respectively. Additionally, a pervious fingerprint-based ML study on FLAP modulators stated that that the reliability of predicted results depends mainly on the compounds themselves rather than algorithms or fingerprints (Tu et al., 2020).

**TABLE 1:** The layout of prediction performances of machine learning models assessed by stratified 5-fold cross-validation for the training set and test set.

Classifier	Training set (*n* = 402)				CV5 of training set (*n* = 402)				Test set (*n* = 101)			
	SE	SP	ACC	MCC	SE	SP	ACC	MCC	SE	SP	ACC	MCC
XGBoost (GBDT)	0.99	0.99	0.99	0.98	0.91	0.89	0.90	0.81	0.89	0.91	0.90	0.80
Random forest (RF)	0.99	1.00	1.00	0.99	0.85	0.90	0.87	0.75	0.94	0.88	0.91	0.82
Decision tree (DT)	0.88	0.94	0.91	0.82	0.83	0.83	0.83	0.66	0.83	0.84	0.84	0.68
Support vector machine (SVM)	0.96	0.98	0.97	0.93	0.84	0.77	0.80	0.61	0.75	0.80	0.78	0.56
Logistic regression (LR)	0.82	0.88	0.85	0.69	0.84	0.75	0.79	0.59	0.85	0.87	0.86	0.72
Multilayer perceptron (MLP)	0.79	0.81	0.80	0.60	0.72	0.78	0.75	0.50	0.70	0.71	0.70	0.40

The lowest performance was shown by the MLP model with an accuracy value of 0.75 and an MCC value of 0.50. For the best model (XGBoost), RFECV curve jumps to a maximum accuracy when the 46 informative features are captured with feature importance values ranging from 0.01 to 0.4. These 46 features mainly belong to eight descriptor categories named topological indices, 2D matrix-based descriptors, 2D autocorrelations, P_VSA-like descriptors, edge adjacency indices, atom-type E-state indices, pharmacophore descriptors, and molecular properties descriptors. All 46 captured features of the best-performing model (XGBoost) along with description and feature importance values are given in Supplementary Table S1. Additionally, 84, 126, 89, and 90 features have been captured by RF, DT, SVM, and LR, respectively, and RFECV curves for all models with optimal number of selected features are illustrated in Supplementary Figure S1. We anticipate that these 46 2D descriptors have the largest impact to differentiate between highly active and least active FLAP inhibitors. Additionally, the tuned hyperparameters for each model can be found in Supplementary Table S2.

### 3.2 LipE and cLogP Calculation

LipE and cLogP demonstrate the druggability of a compound in lead optimization programs to evaluate the potential for better *in vivo* efficacy and safety. A graph between pIC_50_ and cLogP along with LipE values of the compounds in the training set is shown in Supplementary Figure S2. In the current dataset of 658 FLAP inhibitors, only 238 compounds out of 658 demonstrated LipE value greater than five, which is the optimal threshold with cLogP values in the range of −0.27 to 3.78. Moreover, only 136 compounds showed a cLogP value between optimal range of 2–3.5 as proposed by Leeson and Springthorpe (2007). Additionally, 349 compounds out of 658 exhibited values of LipE less than 1 (cLogP = 4.3–10.19) while the cLogP range for 309 compounds having a LipE value greater than one was observed as 0.27–7.88. Interestingly, several potent FLAP inhibitors such as MK-886 (pIC_50_ = 8.65 cLogP = 8.58, LipE = 0.07), MK-591 (pIC_50_ = 9.30 cLogP = 8.82, LipE = 0.48), AM-643 (pIC_50_ = 8.69, cLogP = 7.72, LipE = 0.97), AM-679 (pIC_50_ = 8.65, cLogP = 7.98, LipE = 0.67), AM-803 (pIC_50_ = 8.53, cLogP = 8.97, LipE = 0.43), and BRP-7 (pIC_50_ = 6.50, cLogP = 7.23, LipE = 0.72) displayed significantly low values of LipE. It seems that increase in potency of these compounds might be due to increase in lipophilicity. On the other hand, other FLAP modulators such as BI665915 (pIC_50_ = 8.76, cLogP = 2.14, LipE = 6.62) and AZD6642 (pIC_50_ = 8.31, cLogP = 1.72, LipE = 6.62) showed relatively high values of LipE.

Herein, a dataset of 187 compounds having LipE value greater than one and cLogP greater than two was selected for further application of molecular modeling studies as all FLAP inhibitors in clinical trials possess high values of lipophilicity (cLogP). The dataset of 187 compounds was subsequently divided into a training set (151 compounds, Supplementary Table S3) and a test set (36 compounds, Supplementary Table S4). Docking-guided GRIND analysis was performed on the training set followed by evaluation of the final GRIND model with the test set.

### 3.3 Molecular Docking and SAR-Guided Pose Analysis

**FIGURE 1:**
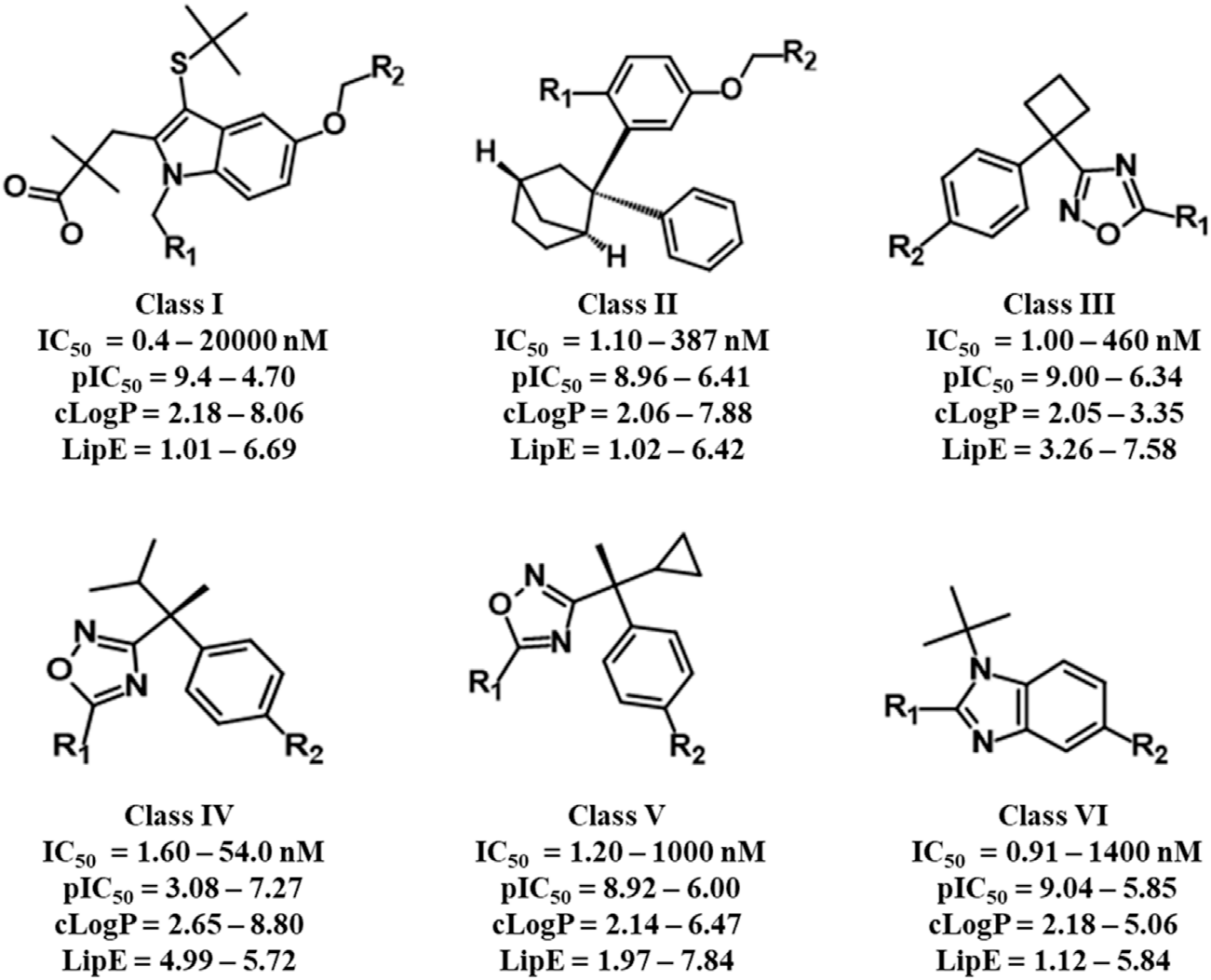
Common scaffolds of six classes of FLAP inhibitors used for common scaffold clustering to obtain the most probable 3D binding poses for employment in GRIND studies.

The selected dataset of 187 compounds mainly consists of already published indoles, biaryl bicycloheptanes, oxadiazole, and benzimidazole-based compounds. The dataset was further divided into a training set (151 compounds) and a test set (36 compounds) and based on common scaffolds; the training set was classified into six distinct classes. Common scaffold along with activity, lipophilicity, and lipophilic efficiency ranges of the six classes is depicted in Figure 1. Furthermore, a binding hypothesis of each class within the FLAP binding cavity was established. The distributions of compounds in each class along with common scaffolds are depicted in Supplementary Table S5.

**FIGURE 2:**
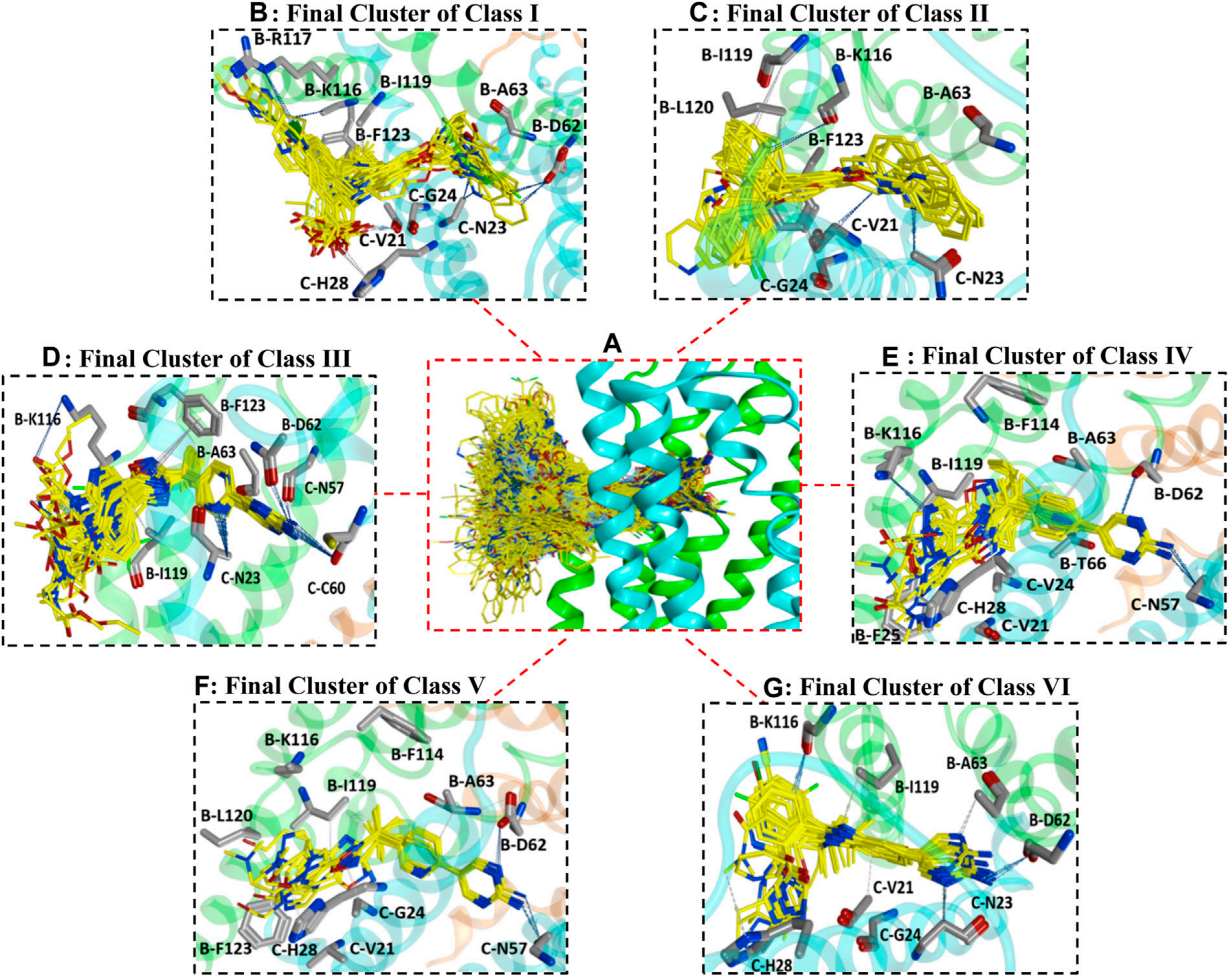
**(A)** illustrates the binding positions and chemical space occupied by all generated poses of 187 FLAP antagonists between chains B and C of the FLAP binding cavity. Chain B is shown in green color, chain C is depicted in blue color, while chain A is depicted in orange color. **(B–G)** represents binding poses of maximum docked ligands in final clusters from class I to VI respectively obtained from common scaffold-based clustering.

All datasets of the selected 187 compounds were docked into the FLAP binding pocket, which included an area of 12 Å selected by assigning x (65.7018), y (58.7512), and z (36.4565) coordinates between chains B and C near previously known interacting amino acid residues (B-F123, B-L120, B-I119, B-R117, B-K116, B-G115, B-F114, B-I113, B-Y112, B-T66, B-A63, B-D62, C-V61, C-C60, C-Q58, C-N57, C-H28, C-A27, C-F25, C-G24, C-N23, and C-V21) (Mancini et al., 1994; Ma et al., 2008). To remove any biases in the docking protocol, 100 poses per ligand were generated using the GOLD score fitness function. Further docking solutions were inspected by algoromatics hierarchical cluster analysis based on root mean square deviation (RMSD) at 3.5 Å of the heavy atoms around a common scaffold. To follow the idea of similar binding mode for similar compounds, only those clusters that comprised the maximum number of docked ligands were selected. Overall, one cluster of binding conformations of compounds in all classes have been identified that contained the maximum number of docked ligands. The final selected cluster of each class and details of common scaffold clustering are depicted in Supplementary Table S5. Briefly, 26 out of 32 compounds for class I, 12 out of 20 for class II, 32 out of 35 for class III, 10 out of 10 for class IV, 13 out of 18 for class V, and 26 out of 36 for class VI were clustered out. Interestingly, the binding position of all final clusters was the same, and they bind between helix 4 (α4) and helix 2 (α2) of chain B and helix 1 (α1) and helix 2 (α2) of chain C, but a distinct binding pattern was observed for each class. The binding region between chains B and C occupied by all generated poses of 187 ligands is shown in Figure 2A.

Briefly, class I compounds are derivatives of indole with dimethyl butanoic acid and S-tert-butyl substituents at positions two and three, respectively, as displayed in Figure 1, while R_1_ at position one and R_2_ at position five are generally occupied by heterogeneous 6-membered cyclic rings. The binding solutions for final cluster (cluster 1, Supplementary Table S5) of compounds in class I showed that dimethylbutanoic acid makes π-H-bond interactions with C-H28 and C-Val21, S-tert-butyl makes π-H-bond interactions with B-L120 and B-F123, while the indole scaffold is primarily involved in making π-H-bond interactions with C-G24 (Figure 2B). The R_1_ substituents show hydrogen bonding with B-D62 and C-N23 and π-H interactions contact with B-A63 and C-N23, whereas N of the pyridine ring of R_2_ shows a strong hydrogen bond with B-A63 while R_2_ substituents show hydrogen bonding with B-R117 and B-K116 (Figure 2B). Overall, compounds of class 1 displayed a positive trend (*R* ^2^ = 0.57) between lipophilicity and inhibitory potency (Supplementary Figure S3) and exhibit a distinct SAR pattern. For instance, compound **1** (IC_50_ = 0.4 nM, Supplementary Table S3) having the highest activity value (cLogP = 8.06, LipE = 1.34) among all the datasets contains 5-methylpyridine at R_1_ and para-fluoro-2-phenylpyridine at R_2_ as depicted in Supplementary Table S6. The final docking solution of compound **1** reveals that the pyridine ring present at R_1_ shows a π-H-bond bonding interaction with the -NH_2_ group of B-R117 (Figure 4). Compound **98** (IC_50_ = 9.0 nM, Supplementary Table S6) has a similar structure to compound **1** except for the absence of terminal 5-methyl on R_1_ and the absence of fluorine on R_2_, rendering it low lipophilic (cLogP = 3.35, LipE = 4.69) and less active.

**FIGURE 3:**
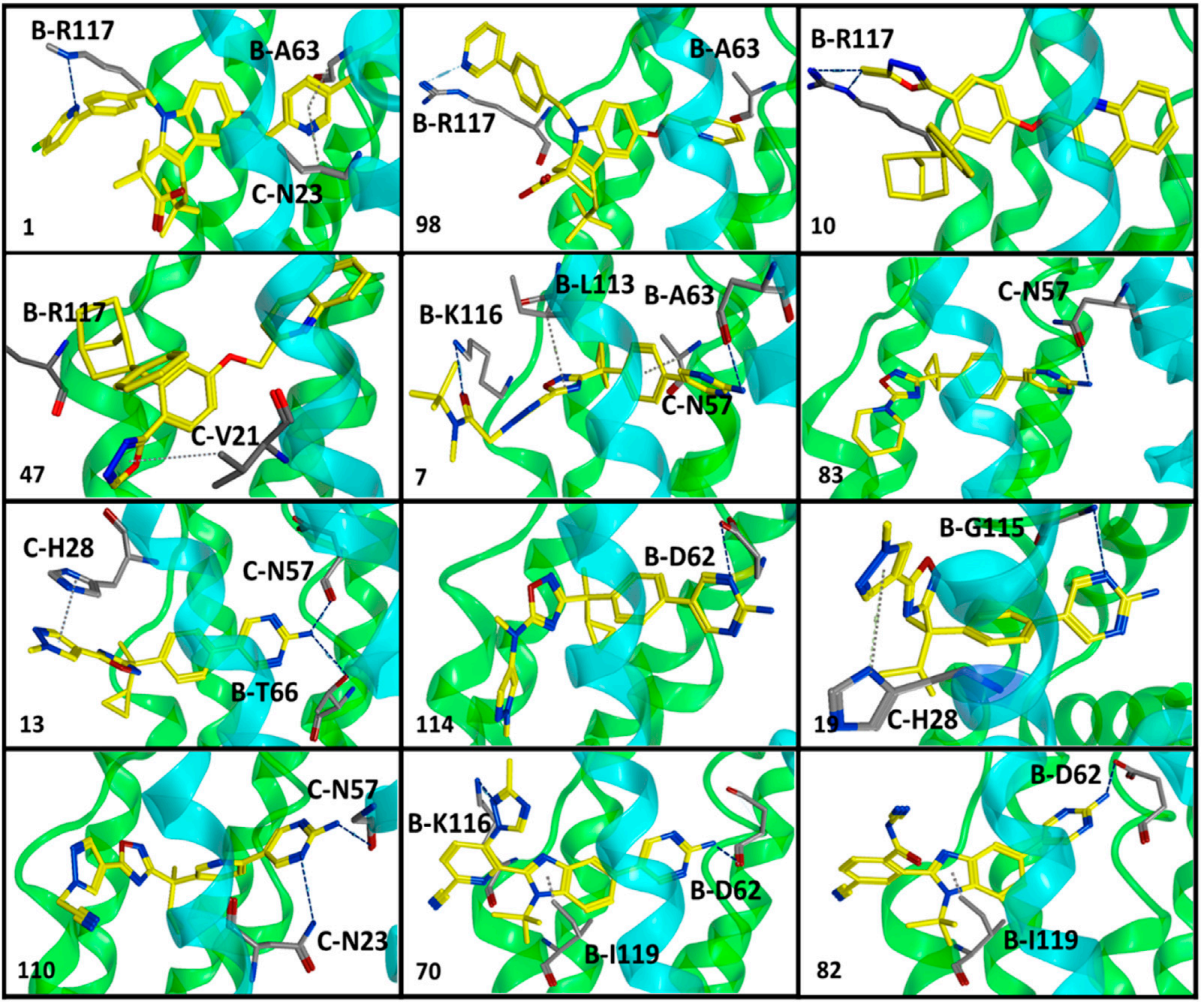
Optimal binding poses of compounds displaying a distinct SAR pattern from all six classes of FLAP modulators. These poses were obtained from clusters with maximum docked ligands (common scaffold clustering) and were further employed for GRID-independent molecular descriptor (GRIND) analysis. Chain B is shown in green while chain C is depicted in blue color.

A study by Stock et al. also established that terminal 5-methyl on pyridine at R_1_ significantly increases the inhibitory potency of compounds against FLAP (N. Stock et al., 2010; N. S. Stock, 2011). Interestingly, the hydrogen bonding between -NH_2_ of B-R117 and nitrogen of the pyridine ring of R_2_ of compound **98** has also been observed in the final docking solutions. However, the pyridine ring at R_1_ did not seem to be involved in making any clear interactions. It was observed that the absence of terminal methyl on the pyridine ring of R_1_ in compound **98** might reduce the exposure of pyridine ring to amino acids inside the FLAP binding cavity (Figure 3), leading to a substantial decrease in inhibitory potency of compound **98**. Moreover, the high LipE value of compound **98** as compared to compound **1** could be attributed only to its low logP (o/w) without an increase in biological activity.

Class II of FLAP antagonists contains 2,2-biaryl bicycloheptane as a common scaffold having diverse substituents at position 2 (R_1_) and quinoline moiety at position 5 (R_2_) of the exo aryl group (Figure 1). Ligand–protein interaction profiling of the final cluster (cluster 3, Supplementary Table S5) shows that the common scaffold orients itself towards the outer side-facing membrane and makes π-H interactions with B-L120, B-I119, and C-V21, whereas the quinoline moiety occupied the inside of the FLAP binding cavity and shows π-H interactions with B-A63, C-G24, and C-N23 (Figure 2D). Generally, R_1_ is involved in making hydrogen bonds with B-F123 and B-K116 amino acid residues. Overall, a slight positive correlation (*R* ^2^ = 0.27) has been observed between inhibitory potency and lipophilicity for class III (Supplementary Figure S3). Moreover, a distinct SAR pattern was observed among compounds of class III. For instance, compound **10** (IC_50_ = 1.1 nM, Supplementary Table S3), being the most potent and lipophilic (cLogP = 7.88, LipE = 1.07) member of this class, possesses oxadiazole-2-thione at R_1_, and absence of thiol substituent of oxadiazole at R_1_ (Supplementary Table S6) resulted in approximately threefold decease in inhibitory potency of compound **47** (IC_50_ = 2.9 nM, cLogP = 7.37, LipE = 1.16). The lipophilicities and LipE values of compounds **10** and **47** are relatively the same and the difference in inhibitory potencies might be due to a distinct binding pattern. The final docking solution of compound **10** reveals the presence of two hydrogen bonds between the terminal sulfur of the oxadiazole-2-thione group at R_1_ and -NH_2_ of B-R117 (Figure 3). In compound **47**, only a π-H-bond interaction was found between the oxadiazole ring of R_1_ and C-V21 that might be not very favorable and contribute to its low inhibitory. The positive contribution of negative ionizable moieties at the oxadiazole ring of R_1_ towards inhibitory potency for class II FLAP antagonists is also evident from previous SAR studies (Chu et al., 2012).

Classes III, IV, and V are cyclobutylbenzene, cyclopropylethylbenzene, and dimethylpropylbenzene derivatives of oxadiazole, respectively (Figure 1). Unlike other three classes, no positive correlation between lipophilicity and inhibitory potency was observed for classes III, IV, and V (Supplementary Figure S3). It means that the difference in inhibitory potency might be due to the distinct interaction pattern and LipE values. All compounds of classes IV, V, and VI contain diverse substituents at R_1_ and pyrimidinamine at R_2_ (Supplementary Table S6), which occupies the inside of the FLAP binding cavity (Figures 2E–G). The final cluster of class III (cluster 3, Supplementary Table S5) reveals that the common scaffold shows π-π stacking with B-F114 and π-H-bond interactions with B-A63 (Figure 2E). R_2_ forms hydrogen bonding with B-D62, C-C60, and π-H-bond interactions with C-N57 and C-N23, while R_1_ seems to be involved in making hydrogen bonds with B-K116 and π-H interactions with B-I119. Compound **7** (IC_50_ = 1.0 nM, cLogP = 3.13, LipE = 5.87, Supplementary Table S3), being the most potent compound of class III, contains N-tert-butyl methylacetamide at the pyrazole ring of R_1_ compared to compound **83** (IC_50_ = 6.5 nM, cLogP = 2.68, LipE = 5.50, Supplementary Table S3), which contains only piperidine ring at R_1_, resulting in a twofold decrease in its inhibitory potency (Supplementary Table S6). The final docking solution of compound **7** reveals that the carbonyl group of N-tert-butyl methylacetamide at R_1_ forms a strong hydrogen bond with -NH_2_ of B-K116 (Figure 3). However, for compound **83**, no interaction was observed between the piperidine ring of R_1_ and amino acid residues of the FLAP binding cavity. The difference in the binding interaction pattern of compounds **7** and **83** might be solely responsible for the difference in inhibitory potencies of both compounds as lipophilicities and LipE values are not significantly different. For class IV, ligand–protein interaction profiling of the final cluster (cluster 4, Supplementary Table S2) suggests that the common scaffold makes π-H interactions with B-T66, B-A63, C-G24, B-I119, and C-Val21, and π-π stacking with B-F114 (Figure 2F). Amino acid residues such as B-D62 and C-N57 present inside the FLAP binding cavity shows hydrogen bonding with R_2_ whereas R_1_ makes hydrogen bonds with B-K116 and π-π interactions with C-H28 and C-F25. Compounds **13** and **114** of class IV were selected to evaluate binding poses due to the distinct SAR pattern (Supplementary Table S6). Compound **13** (IC_50_ = 1.3 nM, cLogP = 2.36, LipE = 6.52, Supplementary Table S3), being the most active from class IV, contains the terminal methyl at the pyrazole ring of R_1_, while compound **114** (IC_50_ = 29.0 nM, cLogP = 4.86, LipE = 2.67, Supplementary Table S3) contains N,1-dimethylpyrazol-4-amine at R_1_, resulting in a twofold decrease in inhibitory potency. The final docking solution of compound **13** reveals that the pyrazole ring of R_1_ is involved in making π-π stacks with C-H28, whereas no significant interaction was observed for terminal methyl (Figure 3). For compound **114**, the final binding pose suggests that the terminal pyrazole ring at R_1_ is unable to show any interactions that might contribute to low inhibitory potency. The significantly low LipE value of compound **114** as compared to compound **13** suggests that gain in activity of compound **13** might be due to its distinct interaction pattern. Similarly, the ligand–protein interaction analysis of the final cluster (cluster 5, Supplementary Table S5) of class V points out that the scaffold makes π-H-bond interactions with B-L120, B-I119, B-A63, C-G24, and C-Val21, and π-π stacks with B-F123 and B-F114 (Figure 2G). R_2_ is involved in making hydrogen bonds with B-D62 and C-N57 while R_1_ makes hydrogen bonds with B-K116 and π-π contact with C-H28. Compound **19** (IC_50_ = 1.6 nM, cLogP = 3.08, LipE = 5.71, Supplementary Table S3) contains terminal methyl at the pyrazole ring of R_1_, whereas compound **110** (IC_50_ = 23 nM, cLogP = 2.65, LipE = 4.98, Supplementary Table S3) possesses acetonitrile at the pyrazole ring of R_1_ (Supplementary Table S6) and approximately two orders of magnitude decrease of inhibitory potency was observed for compound **110** as compared to compound **19**. The final binding pose of compound **19** reveals that the pyrazole ring of R_1_ is involved in making π-π contact with C-H28 while terminal methyl could not make any interactions (Figure 3). The absence of interactions between acetonitrile at R_1_ of compound **110** and amino acid residues of the FLAP binding pocket was likely the reason for the two orders of magnitude decrease in inhibitory potency of compound **110** as LipE and lipophilicity values of both compounds do not differ appreciably. Overall, compounds of classes III, IV, and V displayed better LipE values, but the high inhibitory potencies of highly active compounds are due to strong interactions among particular functional groups and amino acids of the FLAP binding cavity.

Class VI FLAP inhibitors are benzimidazole derivatives (Figure 1) having diverse substituents at R_1_ and pyrimidinamine at R_2_ around the benzimidazole scaffold. The compounds of class VI did not exhibit any correlation between activity and lipophilicity (Supplementary Figure S3). The ligand–protein interaction profile of the final cluster indicated that the pyrimidinamine group orients itself towards the inner side of the FLAP binding cavity and is involved in making hydrogen bonds with B-D62 and C-N23, whereas the common scaffold occupies the between chains B and C and forms π-H bonding with B-I119, C-G24, and C-V21. The diverse R_1_ is involved in making hydrogen bonds with B-K116 and π-π interactions with C-H28. Compounds of class VI showed a distinct SAR pattern; e.g., in compound **70** (IC_50_ = 4.2 nM, cLogP = 2.54, LipE = 5.84), the pyridine moiety of R_1_ contains methyl triazole at position three and its replacement with acetonitrile in compound **82** resulted in two orders of magnitude decrease in the inhibitory potency of compound **82** (IC_50_ = 6.09 nM, cLogP = 2.18, LipE = 6.04). The selected binding pose of compound **70** indicated that the triazole ring is making hydrogen bonds with B-K116 whereas no interaction was observed between acetonitrile and amino acid residues of the FLAP binding cavity in the case of compound **82** (Figure 3). The compounds of class VI did not show any correlation with LipE, which means that the difference in binding interactions is the main driving factor behind the difference in activity.

Overall, our criteria for the selection of compounds for molecular modeling studies were cLogP and LipE. However, our results indicate that only cLogP contributes slightly positively towards inhibitory potency for classes I and II, whereas for compounds of classes III, IV, V, and VI, the difference in interaction pattern might be exclusively responsible for the difference in inhibitory potency, as in these classes of FLAP inhibitors, the high LipE values were maintained due to loss in lipophilicity. In addition, our docking results suggest that heterocyclic moieties are involved in making π-H interactions with hydrophobic amino acid residues of the FLAP binding cavity. Therefore, the presence of pyridine, pyrimidine, pyridazine, pyrazole, triazole, and oxadiazole rings moderately increases not only the lipophilicity but also the inhibitory potency. Moreover, an increase or decrease in LipE values of FLAP inhibitors does not alter the inhibitory potencies in either way. Further docking poses obtained from multiple clusters with maximum docked ligands were employed to generate the vGRIND model.

### 3.4 GRID-Independent Molecular Descriptors Analysis

**FIGURE 4:**
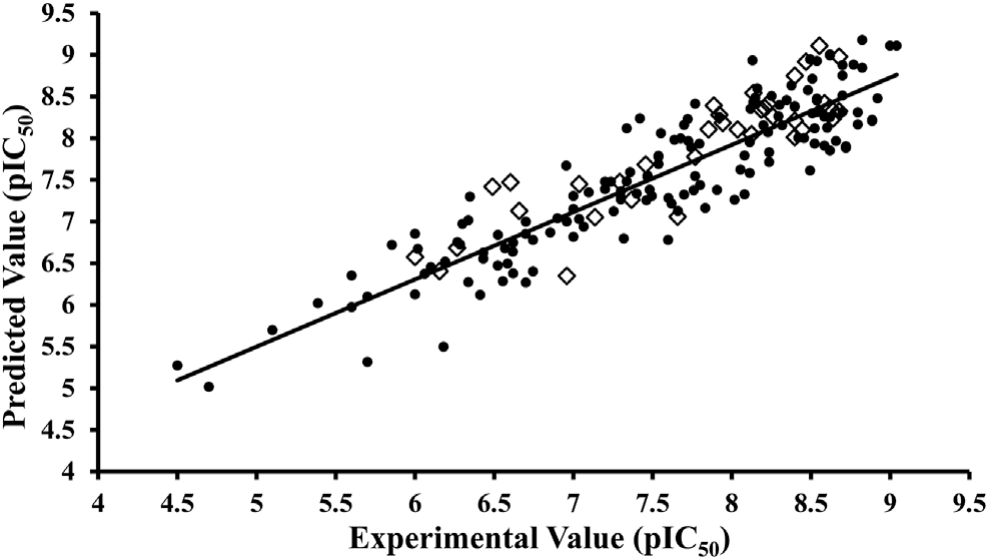
Activity interactive graph plot between predicted and actual experimental activity values. The graph plot displays separate data series for training (filled circles) and test (rhombus) set. *R* ^2^ for training set was observed as 0.82 and 0.77 for test set.

The selected binding poses of 151 (Supplementary Table S3) compounds of the training set obtained through common scaffold clustering of docking poses along with their inhibitory activity (pIC_50_) values were implied in the pentacle v 1.07 software package that utilizes special alignment independent GRIND descriptors to develop a 3D-QSAR model. To correlate the inhibitory potencies with 3D structural features and to derive the most important pharmacophoric features of our training set, a partial least square model was developed on five principal components using the leave-one-out (LOO) cross-validation method, resulting in initial models with satisfactory values of variables. The inconsistent nodes were removed by one-time application of the fractional factorial design (FFD) variable selection algorithm. The final GRIND model was obtained with good values of performance measures, *q* ^*2*^ = 0.66 and *R* ^2^ = 0.82, while the standard error of prediction (SDEP) was 0.47. The before and after FFD application statistics along with the r^2^m metric is shown in Table 2. The difference between actual and predicted activity values was less than one log unit for all 151 inhibitors of the training set as shown in Figure 4. The test set (Supplementary Table S4) was used for the evaluation of the final GRIND model, which predicted inhibitory potencies of test set compounds with a difference of less than one log unit for all compounds between experimental and predicted pIC_50_ values with *R* ^*2*^ value observed as 0.77 (Figure 4).

**TABLE 2:** Statistical parameters obtained before and after application of fractional factorial design (FFD) on final GRIND model.

Fractional factorial design cycle (FFD)											
Complete variable						FFD1					
Datasets	*R* ^2^	$q_{LOO}^{2}$	SDEP	$r_{m}^{2}$	Delta $r_{m}^{2}$	Datasets	*R* ^2^	$q_{LOO}^{2}$	SDEP	$r_{m}^{2}$	Delta $r_{m}^{2}$
Training set	0.71	0.60	0.49	0.703	0.004	Training set	0.82	0.66	0.47	0.775	0.001
Test set	0.63	0.58	0.49	0.517	0.028	Test set	0.77	0.64	0.47	0.686	0.012

**FIGURE 5:**
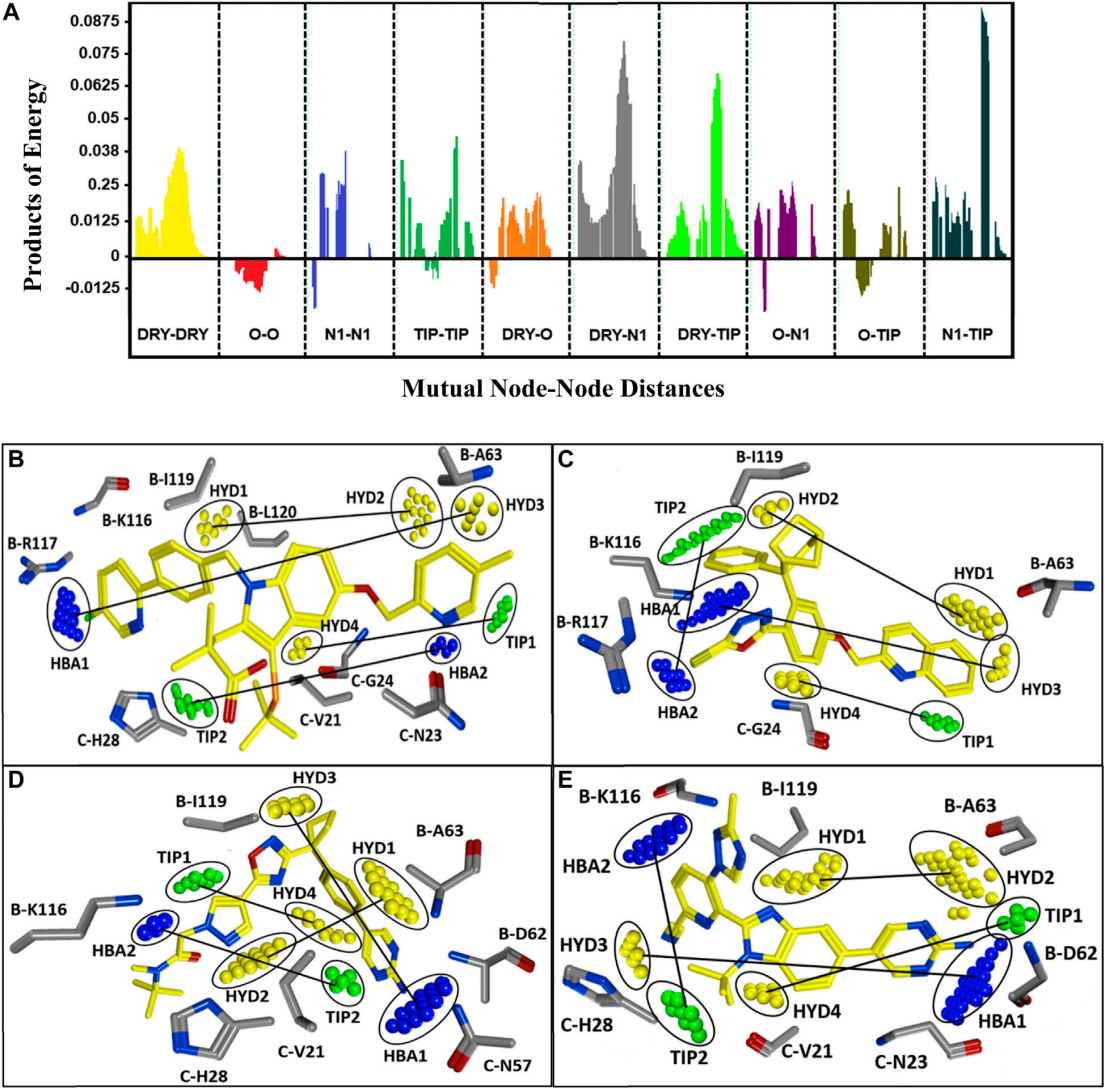
**(A)** Correlogram of PLS coefficients representing the pair of probes contributing positively (peaks above 0) or negatively (peaks below 0) towards the inhibitory potencies of training set compounds. The positive contribution towards pIC_50_ of FLAP inhibitors has been depicted by DRY-DRY (two hydrophobic), DRY-N1 (one hydrophobic and one hydrogen bond acceptor), DRY-TIP (one hydrophobic and one steric), and N1-TIP (one hydrogen bond acceptor and one steric) variables. The variables are present in all highly active FLAP compounds and are located at mutual distances of 16.00–16.40 Å, 16.40–16.80 Å, 18.00–18.40 Å, and 17.20–17.60 Å, respectively. **(B)** The identified hotspots on most active indole-based FLAP inhibitor (compound 1) of training set with projection of actual FLAP structure. Hydrophobic features are depicted in yellow, hydrogen bond acceptors are in blue, while steric hotspots are depicted in green color. The two hydrophobic hotspots (HYD1 and HYD2) are located between two aromatic moieties, one hydrophobic (HYD3) and one hydrogen bond acceptor feature (HBA1) are present between aromatic rings and terminal negative ionizable substitution, one hydrophobic (HYD4) and steric feature (TIP1) can be spotted between aromatic ring and indole scaffold, while one hydrogen bond acceptor (HBA2) and one steric (TIP2) hotspot are present between dimethylbutanoic acid and pyridine ring. **(C)** The most active compound (compound 10) of class II with mapping of complemented amino acids on the recognized contours. **(D)** The most active of class III (compound 7), which is also the most active compound from oxadiazole-based FLAP antagonists (classes III, IV, and V) and mapped hotspots along with projection of complementary amino acids of FLAP binding cavity. Due to high structural similarity, the features were also observed at the same positions in all active compounds of classes IV and V. **(E)** The compound (70) from class VI with identified hotspots and corresponding amino acids.

A PLS coefficient correlogram of the GRIND variables is shown in Figure 5A and describes important 3D structural features that directly/inversely correlate with the inhibitory potencies of the training set compounds. The PLS coefficient correlogram depicts that DRY-DRY, DRY-N1, DRY-TIP, and N1-TIP pair of probes positively contribute towards the inhibitory potency of chemically diverse FLAP inhibitors whereas no inverse contribution was observed by any variable. These variables are located at a certain distance within active inhibitors between substitutions at R_1_, R_2_, and common scaffolds.

More explicitly, the DRY-DRY correlogram in Figure 5A shows the presence of two hydrophobic contours (HYD1 and HYD2) at a mutual distance of 16.00–16.40 Å in a virtual receptor site of highly active FLAP inhibitors pIC_50_ > 7.5. For class I, the distance is present between the pyridine ring of R_1_ and the phenyl ring of R_2_; for class II, it is observed between the quinoline group and the endo aryl moiety of the common scaffold; for classes III, IV, and V, it is present between the pyrazole ring at R_1_ position and the phenyl ring of the common scaffold; and for class VI, it was observed between phenyl of the common scaffold and pyrimidinamine of R_2_ (Figures 5B–E). Furthermore, the backstage projection of the actual FLAP structure onto the identified hotspots revealed the presence of complementary hydrophobic amino acid residues such as B-A63, B-I119, and B-L120. This further strengthened our docking outcomes as all of these amino acid residues are involved in making extensive π-H interactions with dataset compounds. Additionally, a recent pharmacophore study of Olgac et al. revealed that four hydrophobic features are important in most potent indole- and oxadiazole-based FLAP inhibitors (Olgac et al., 2020).

Similarly, DRY-N1 (Figure 5A) explicates the positive contribution of one hydrophobic (HYD3) and one hydrogen bond acceptor (HBA1) at a mutual distance of 16.40–16.80 Å within active FLAP modulators. Interestingly, this distance was observed in all highly active FLAP modulators pIC_50_ > 7.5 and absent in all less-active compounds pIC_50_ < 7.5. Briefly, for class I, it is observed between the terminal negative ionizable moiety present at R_2_ and the pyridine ring of R_1_; in class II, it is observed between the quinoline group and pyrazole ring; for classes III, IV, and V, it is present between the pyrimidinamine group of R_2_ and oxadiazole ring; and for class VI, it was observed between pyrimidinamine and pyridine of R_1_ as displayed in Figures 5B–E. Projecting actual FLAP structure onto the identified virtual hotspots revealed the presence of hydrophobic amino acids B-F114, B-A63, and C-G24 and complementary amide groups in the B-K116 and B-R117 amino acid residues within the FLAP binding cavity that further complements the accuracy of our model. These results further reinforce our docking outcomes, which demonstrated the importance of B-A63 and B-K116 for the hydrophobic and hydrogen bonding interactions within the FLAP binding cavity. These outcomes are also in accord with another pharmacophore-based study that demonstrated the importance of hydrophobic and hydrogen bond acceptor features in the highly active indole- and biaryl bicycloheptane-based FLAP inhibitors (Temml et al., 2017).

Moreover, DRY-TIP correlogram (Figure 5A) portrays the presence of one hydrophobic (HYD4) and one shape-based feature (TIP1) that positively contribute towards the inhibitory potency of FLAP inhibitors. For the sharpest peak, the two contours are present at a mutual distance of 18.00–18.40 Å, indicating the distance between indole scaffold and R_1_ for class I; exoaryl of common scaffold and quinoline for class II; phenyl ring of scaffold and pyrazole ring of R_1_ for classes III, IV, and V; and pyrazole of common scaffold and pyrimidinamine for class VI (Figures 5B–E). Two identified contours (HYD4 and TIP) were mapped on the actual FLAP binding site, and interestingly, the hydrophobic region in all active compounds is complementary to hydrophobic amino acids C-V21, C-G24, and C-H28. It is also in accordance with our docking findings where many compounds of our dataset are involved in making π-H interactions and π-π stacking with these amino acids. The green contour elucidates a steric hotspot region, and it defines the 3D molecular shape of FLAP inhibitors.

The last selected peak N1-TIP (Figure 5A) represents the presence of one hydrogen bond acceptor (HBA2) and one shape-based feature (TIP2) at a mutual distance of 17.20–17.60 Å within highly active FLAP inhibitors. The two features at this distance contributes positively towards the inhibitory potency of compounds against FLAP. The hydrogen bond acceptor hotspot in the virtual receptor site of FLAP is complemented by the presence of B-K116, B-D62, C-N57, and C-N23 amino acids in the actual receptor site when we mapped the FLAP structure onto the identified N1 (HBA2) hotspot. The -NH2 and carbonyl groups of these amino acids are involved in making hydrogen bonds with active FLAP modulators as evident from our docking studies and pose analysis. Moreover, these features have been observed in all active FLAP inhibitors pIC_50_ > 7.5 while they are absent in less active compounds pIC_50_ < 7.5. For class I, the distance is present between the pyrimidine ring at R_2_ position and dimethylbutanoic acid; for class II it is observed between substituents at the pyrazole ring of R_1_ and endo aryl of scaffold; for classes III, IV, and V, it is present between the pyrimidine amine of R_2_ and pyrazole ring at R_1_ position; and for class VI, it is present between the triazole of R_1_ and tertbutyl of the common scaffold (Figures 5B–E). The TIP probe signifies the importance of a steric hotspot at a distance of 17.20–17.60 Å from the hydrogen bond acceptor feature.

Generally, our study provided a deeper understanding of three-dimensional requirements of diverse inhibitor binding within the FLAP binding cavity by mapping the mutual distances of important pharmacophoric features (four hydrophobic, two hydrogen bond acceptor, and two steric features) as well as the complementary distances of the important interacting amino acid residues (B-L120, B-I119, B-R117, B-K116, B-F114, B-A63, B-D62, C-H28, C-G24, C-N23, and C-V21). Previous docking studies also revealed that highly potent FLAP modulators result in π-π stacking with C-H28, hydrophobic interactions with B-L120, B-I119, and hydrogen bonding with B-R117, B-K116, and B-D62 (Ma et al., 2008). Overall, the binding hypothesis generated for each class within the FLAP binding cavity was complementary with our GRIND model, which predicted the inhibitory potencies of validation and test sets with reasonable accuracy, indicating the fitness of our model. Based on our current findings, we suggest that the high inhibitory potency of a compound against FLAP can be achieved by (1) increasing the hydrogen bond acceptor strength on at least one substitution position (R_1_ or R_2_); (2) insertion of heterocyclic moieties such as pyridine, pyrimidine, pyridazine, pyrazole, and triazole at each side of the common scaffold to increase hydrophobic strength; and (3) maintaining a distance of 16.00–16.40 Å between two hydrophobic groups (aromatic rings) and 16.40–16.80 Å between hydrophobic and hydrogen bond acceptor groups.

## 4 Discussion

Since high levels of leukotrienes have been reported in multiple pathophysiological conditions in the past 3 decades, leukotriene synthesis pathway has been targeted at many levels while FLAP has received the greatest focus because it initiates the biological synthesis of leukotrienes *via* leukotriene synthesis pathway (Massoumi and Sjölander 2007; Bryda and Wątroba 2018; Jo-Watanabe, Okuno, and Yokomizo 2019). Several practices have been made to propose potent FLAP modulators, and many of them have shown good clinical efficacy. However, not a single molecule could be able to change into the status of “drug”. The focus of the present study is to unveil the two- and three-dimensional structural requirements of FLAP modulators.

First to demonstrate the important two-dimensional structural features, supervised ML approach was adapted over classical 2D QSAR modeling. The preference was made for two reasons: (1) to escape the alignment step as FLAP modulators are highly diverse in nature, and (2) evidence from the past strengthens the adaptation of ML for quantitative structure–activity relationship studies (Tsou et al., 2020; Gupta et al., 2021). We developed multiple ML models including XGboost (GBDT), random forest (RF), decision tree (DT), support vector machine (SVM), logistic regression (LR), and multilayer perceptron (MLP), and in comparison, XGBoost and RF were able to classify our training set and predict the test set with significant classification and prediction accuracies. Moreover, recursive feature elimination with cross-validation (RFECV) captured relevant features or 2D descriptors, which are mainly participating in the classification of highly active and least active FLAP inhibitors.

Further molecular modeling studies were performed to vaticinate the important three-dimensional pharmacophoric features instead of ML. The preference was made because (1) three-dimensional structural properties are highly dependent on binding poses, and (2) the GRIND model not only explains the important molecular interaction fields but also distances between them along with important amino acid residues by creating a virtual receptor site (Shafi and Jabeen 2017). Before implication of molecular modeling strategies, the dataset was first subjected to calculation of LipE and cLogP. The purpose of LipE-based lead optimization is to improve LipE while maintaining an appropriate range of logP for the optimization of potency and ADME properties. The increased potency of a compound with eque-LipE to the reference ligand demonstrates that the increase in lipophilicity alone is responsible for the increased potency, although other factors associated with the specific structural change cannot be ruled out. Also, an increase in LipE of a compound suggests that an increase in potency is beyond lipophilicity increases alone and other factors such as transport to the target and hydrogen bonding strength within the protein binding site could be associated with this response. In total, 238 out of 658 demonstrated the LipE value greater than five, which is the optimal threshold, and only 136 demonstrated cLogP between the optimal range of 2–3.5. Herein, we selected 187 compounds having LipE greater than one and cLogP greater than two because FLAP is an integral membrane protein, which means that compounds should possess a high lipophilicity value for efficient binding. Also, FLAP modulators in clinical trials usually possess lipophilicity >3. The dataset of 187 compounds was divided into six distinct classes based on the common scaffold with subsequent docking into the FLAP binding pocket. Our docking results indicated that the FLAP binding pocket can cater diverse anti-inflammatory compounds and they bind between chains B and C. The ligand–protein interaction profile of selected FLAP modulators revealed that mostly B-R117, BK-116, C-N57, C-N23, and B-D62 FLAP amino acid residues are involved in making hydrogen bonds; B-A63, B-L119, B-L120, B-V21, and C-G24 make π-H-bond interactions, whereas C-H28 is involved in forming π-π contact with FLAP modulators. Also, for classes I and II, a moderate correlation was observed between lipophilicity and inhibitory potency, whereas for compounds of classes III, IV, V, and VI, an increase or decrease in lipophilicity or LipE did not alter the inhibitory potency in either way or *vice versa*.

To select the most probable binding poses, common scaffold clustering was performed because using GRID-independent molecular descriptors, analysis of 3D structural features is highly dependent on 3D confirmations of the molecules (Pastor et al., 2000). Multiple clusters at 3.5 Å RMSD were generated and binding poses from clusters with maximum number of docked ligands were further used to build the GRIND model. The reliability of binding pose selection *via* common scaffold clustering for generation of the GRIND model can be explained by satisfactory statistical results obtained for the final GRIND model. Furthermore, the model signifies the positive contribution of four hydrophobic, two hydrogen bond acceptor, and two steric features towards the inhibitory potency of FLAP modulators. The identified hotspots or pharmacophoric features were successfully mapped onto the highly active FLAP modulators followed by projection of the actual receptor site, which revealed the presence of corresponding amino acid residues. Overall, our GRIND model suggested that (1) two hydrophobic features should be present at a mutual distance of 16.00–16.40 Å, (2) one hydrophobic and hydrogen bond acceptor feature should be present at a distance of 16.40–16.80 Å, (3) the distance between hydrophobic and steric feature should be 18.00–18.40 Å, and (4) and it should be 17.20–17.60 Å between hydrogen bond acceptor and steric features. The importance of hydrophobic and hydrogen bond acceptor features has also been demonstrated by previous studies (Temml et al., 2017; Olgac et al., 2020).

Based on these findings, further analyses will focus on virtual screening from both ML and GRIND models followed by selection of common compounds. The common hits can further be structurally tuned and optimized. The ML and GRIND model will allow internal inspection of FLAP modulators, before validating them using predictions on vendor libraries, purchase, and testing.

## 5 Conclusion

The current study deals with the development of ML models and a GRIND model on a diverse series of FLAP inhibitors. First of all, our ML models signify some important 2D descriptors, and the best-performing model (XG-Boost) has successfully classified the active and inactive compounds present in our training set exhibiting 91% overall classification accuracy. The subsequent screening of test set from the model resulted in 90% prediction accuracy, which further accentuates the efficiency of the model. Secondly, docking studies reveal that hydrogen bonding and hydrophobic interactions are critical for binding of FLAP inhibitors. Further common scaffold-based clustering revealed the optimal binding mode of structurally diverse inhibitors and aided in determination of their molecular basis of interaction within the FLAP binding cavity. Thirdly, the most probable binding poses were utilized for GRIND model development, which showed valid statistical results having an *R* ^2^ of 0.82 and a *q* ^2^ of 0.66. Additionally, the GRIND model predicted all compounds of training and test set with an activity difference of less than one log unit. Overall, our GRIND model illustrated that four hydrophobic, two hydrogen bond acceptor, and two steric features are critical for achieving high inhibitory potency against FLAP. All the features were successfully complemented by the docking studies highlighting the significance of respective amino acid residues such as B-L120, BI119, B-A63, C-H28, C-G24, and C-V21 for hydrophobic interactions and B-R117, B-K116, D-62, and C-N57 for hydrogen bonding. In general, application of ML, docking analysis, common scaffold clustering, and GRIND modeling to predict the 2D structural requirements as well as the 3D molecular basis of interaction of diverse FLAP inhibitors could potentially guide the development of more potent chemotypes for the treatment of inflammatory disorders requiring anti-leukotriene therapy.

## Data Availability

The original contributions presented in the study are included in the article/Supplementary Material. Further inquiries can be directed to the corresponding author.
