# Supplementary material for: Combined Machine Learning and GRID-Independent Molecular Descriptor (GRIND) Models to Probe the Activity Profiles of 5-Lipoxygenase Activating Protein Inhibitors
Source: Front Pharmacol. 2022 Mar 1;13:825741. doi: 10.3389/fphar.2022.825741 (PMC8921698; doi:10.3389/fphar.2022.825741)
Supplement: Supplementary file 1 [file DataSheet1.docx]

**SUPPORTING INFORMATION**

**Table S1: Features selected by RFECV with relatively high importance with XGBoost as estimator.**

| # | **Descriptor Name** | **Description** | **Category** | **Importance Value** |
| --- | --- | --- | --- | --- |
| **1** | PW3 | path/walk 3 – Randic shape index | Topological indices | 0.110847 |
| **2** | LOC | lopping centric index | Topological indices | 0.0662145 |
| **3** | IC2 | Information Content index (neighborhood symmetry of 2-order) | Information indices | 0.0519708 |
| **4** | WiA_D/Dt | average Wiener-like index from distance/detour matrix | 2D matrix-based descriptors | 0.0785878 |
| **5** | SM5_B(m) | spectral moment of order 5 from Burden matrix weighted by mass | 2D matrix-based descriptors | 0.1821463 |
| **6** | ChiA_B(v) | average Randic-like index from Burden matrix weighted by van der Waals volume | 2D matrix-based descriptors | 0.2917202 |
| **7** | VE2sign_B(e) | average coefficient of the last eigenvector from Burden matrix weighted by Sanderson electronegativity | 2D matrix-based descriptors | 0.1220707 |
| **8** | SM6_B(p) | spectral moment of order 6 from Burden matrix weighted by polarizability | 2D matrix-based descriptors | 0.1365065 |
| **9** | MATS4v | Moran autocorrelation of lag 4 weighted by van der Waals volume | 2D autocorrelations | 0.0572592 |
| **10** | MATS3e | Moran autocorrelation of lag 3 weighted by Sanderson electronegativity | 2D autocorrelations | 0.0715563 |
| **11** | MATS6e | Moran autocorrelation of lag 6 weighted by Sanderson electronegativity | 2D autocorrelations | 0.07488206 |
| **12** | MATS8e | Moran autocorrelation of lag 8 weighted by Sanderson electronegativity | 2D autocorrelations | 0.43728649 |
| **13** | MATS2p | Moran autocorrelation of lag 2 weighted by polarizability | 2D autocorrelations | 0.2773454 |
| **14** | MATS2i | Moran autocorrelation of lag 2 weighted by ionization potential | 2D autocorrelations | 0.0970533 |
| **15** | MATS4i | Moran autocorrelation of lag 4 weighted by ionization potential | 2D autocorrelations | 0.07623153 |
| **16** | GATS5v | Geary autocorrelation of lag 5 weighted by van der Waals volume | 2D autocorrelations | 0.11131419 |
| **17** | GATS2e | Geary autocorrelation of lag 2 weighted by Sanderson electronegativity | 2D autocorrelations | 0.09690023 |
| **18** | GATS2p | Geary autocorrelation of lag 2 weighted by polarizability | 2D autocorrelations | 0.01949001 |
| **19** | JGI4 | mean topological charge index of order 4 | 2D autocorrelations | 0.01876386 |
| **20** | JGI10 | mean topological charge index of order 10 | 2D autocorrelations | 0.05998653 |
| **21** | P_VSA_MR_5 | P_VSA-like on Molar Refractivity, bin 5 | P_VSA-like descriptors | 0.069783 |
| **22** | P_VSA_m_2 | P_VSA-like on mass, bin 2 | P_VSA-like descriptors | 0.01704044 |
| **23** | P_VSA_ppp_ter | P_VSA-like on potential pharmacophore points, ter – terminal atoms | P_VSA-like descriptors | 0.03396779 |
| **24** | P_VSA_charge_1 | P_VSA-like on partial charges, bin 1 | P_VSA-like descriptors | 0.01104589 |
| **25** | P_VSA_charge_6 | P_VSA-like on partial charges, bin 6 | P_VSA-like descriptors | 0.1445477 |
| **26** | P_VSA_charge_9 | P_VSA-like on partial charges, bin 9 | P_VSA-like descriptors | 0.2127567 |
| **27** | Eta_L_A | eta average local composite index | ETA indices | 0.0152341 |
| **28** | Eta_sh_y | eta p shape index | ETA indices | 0.00673492 |
| **29** | SpMAD_EA(ri) | spectral mean absolute deviation from edge adjacency mat. weighted by resonance integral | Edge adjacency indices | 0.01393901 |
| **30** | Chi1_EA(dm) | connectivity-like index of order 1 from edge adjacency mat. weighted by dipole moment | Edge adjacency indices | 0.01537356 |
| **31** | SM04_EA(dm) | spectral moment of order 4 from edge adjacency mat. weighted by dipole moment | Edge adjacency indices | 0.2158478 |
| **32** | SM13_AEA(ed) | spectral moment of order 13 from augmented edge adjacency mat. weighted by edge degree | Edge adjacency indices | 0.1416984 |
| **33** | Eig04_AEA(ed) | eigenvalue n. 4 from augmented edge adjacency mat. weighted by edge degree | Edge adjacency indices | 0.09672215 |
| **34** | SaasC | Sum of aasC E-states | Atom-type E-state indices | 0.02524075 |
| **35** | minaasC | Mimimum aasC | Atom-type E-state indices | 0.11350487 |
| **36** | MaxaasC | Maximum aasC | Atom-type E-state indices | 0.34275624 |
| **37** | MaxssssC | Maximum ssssC | Atom-type E-state indices | 0.10982074 |
| **38** | CATS2D_04_AL | CATS2D Acceptor-Lipophilic at lag 04 | Pharmacophore descriptors | 0.04664152 |
| **39** | CATS2D_05_AL | CATS2D Acceptor-Lipophilic at lag 05 | Pharmacophore descriptors | 0.04672782 |
| **40** | CATS2D_01_LL | CATS2D Lipophilic-Lipophilic at lag 01 | Pharmacophore descriptors | 0.2638778 |
| **41** | SHED_LL | SHED Lipophilic-Lipophilic | Pharmacophore descriptors | 0.3207752 |
| **42** | SHED_DA | SHED Donor-Acceptor | Pharmacophore descriptors | 0.11503894 |
| **43** | F05[C-N] | Frequency of C – N at topological distance 5 | 2D Atom Pairs | 0.0127567 |
| **44** | Uc | unsaturation count | Molecular properties | 0.02004674 |
| **45** | SAscore | Synthetic Accessibility score | Molecular properties | 0.01685369 |
| **46** | MDEC-33 | molecular distance edge between all tertiary carbons | MDE descriptors | 0.05623682 |

**Table S2: Tuned parameters for final machine learning models trained in this work.**

| **Model Name** | **Optimal Hyperparameters** |
| --- | --- |
| XGBoost | *base_score=*0.5*, booster=*'gbtree'*, colsample_bylevel=*1*, colsample_bynode=*1*, colsample_bytree=*1*, gamma=*0*, learning_rate=*0.1*, max_delta_step=*0, *max_depth=*5*, min_child_weight=*3*, missing=*None, *n_estimators=*200, *n_jobs=*1, *nthread=*None, *objective=*'binary’, *random_state=*0, *reg_alpha=*0, *reg_lambda=*1, *scale_pos_weight=*1, *seed=*None, *silent=*None, *subsample=*1, *verbosity=*1 |
| RF | *n_estimators*=150, *criterion*='gini', *max_depth*=None, *min_samples_split*=10, *min_samples_leaf*=1, *min_weight_fraction_leaf*=0.0, *max_features*='sqrt', *max_leaf_nodes*=None, *min_impurity_decrease*=0.0, *min_impurity_split*=None, *bootstrap*=True, *oob_score*=False, *n_jobs*=None, *random_state*=None, *verbose*=0, *warm_start*=False, *class_weight*=None, *ccp_alpha*=0.0, *max_samples*=None |
| SVM | *C*=11, *kernel*='linear', *degree*=3, *gamma*='scale', *coef0*=0.0, *shrinking*=True, *probability*=False, *tol*=0.001, *cache_size*=200, *class_weight*=None, *verbose*=False, *max_iter*=- 1, *decision_function_shape*='ovr', *break_ties*=False, *random_state*=None |
| DT | *criterion*='gini', *splitter*='best', *max_depth*=None, *min_samples_split*=2, *min_samples_leaf*=1, *min_weight_fraction_leaf*=0.0, *max_features*=70, *random_state*=None, *max_leaf_nodes*=None, *min_impurity_decrease*=0.0, *min_impurity_split*=None, *class_weight*=None, *ccp_alpha*=0.0 |
| LR | *penalty*='l1', *dual*=False, *tol*=0.0001, *C*=1.0, *fit_intercept*=True, *intercept_scaling*=1, *class_weight*=None, *random_state*=None, *solver*='lbfgs', *max_iter*=100, *multi_class*='auto', *verbose*=0, *warm_start*=False, *n_jobs*=None, *l1_ratio*=None |
| MLP | *hidden_layer_sizes*=(150, 100), *activation*='relu', *solver*='adam', *alpha*=0.0001, *batch_size*='auto', *learning_rate*='constant', *learning_rate_init*=0.001, *power_t*=0.5, *max_iter*=200, *shuffle*=True, *random_state*=None, *tol*=0.0001, *verbose*=False, *warm_start*=False, *momentum*=0.9, *nesterovs_momentum*=True, *early_stopping*=False, *validation_fraction*=0.1, *beta_1*=0.9, *beta_2*=0.999, *epsilon*=1e-08, *n_iter_no_change*=10, *max_fun*=15000 |


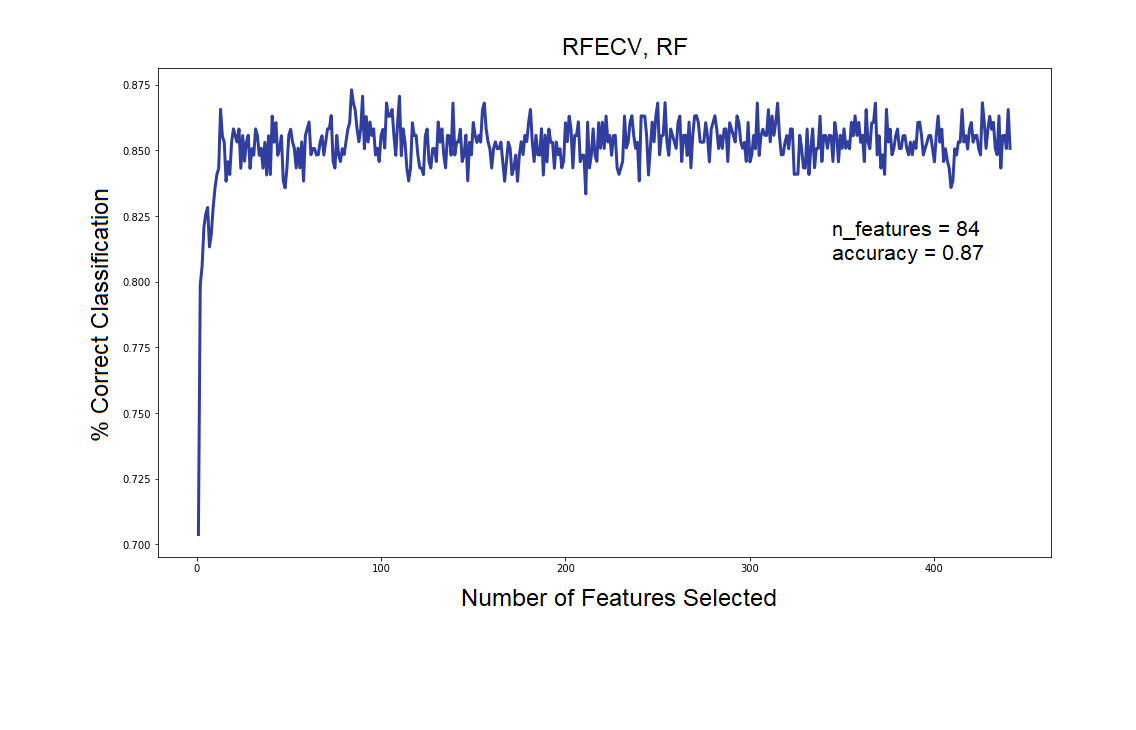

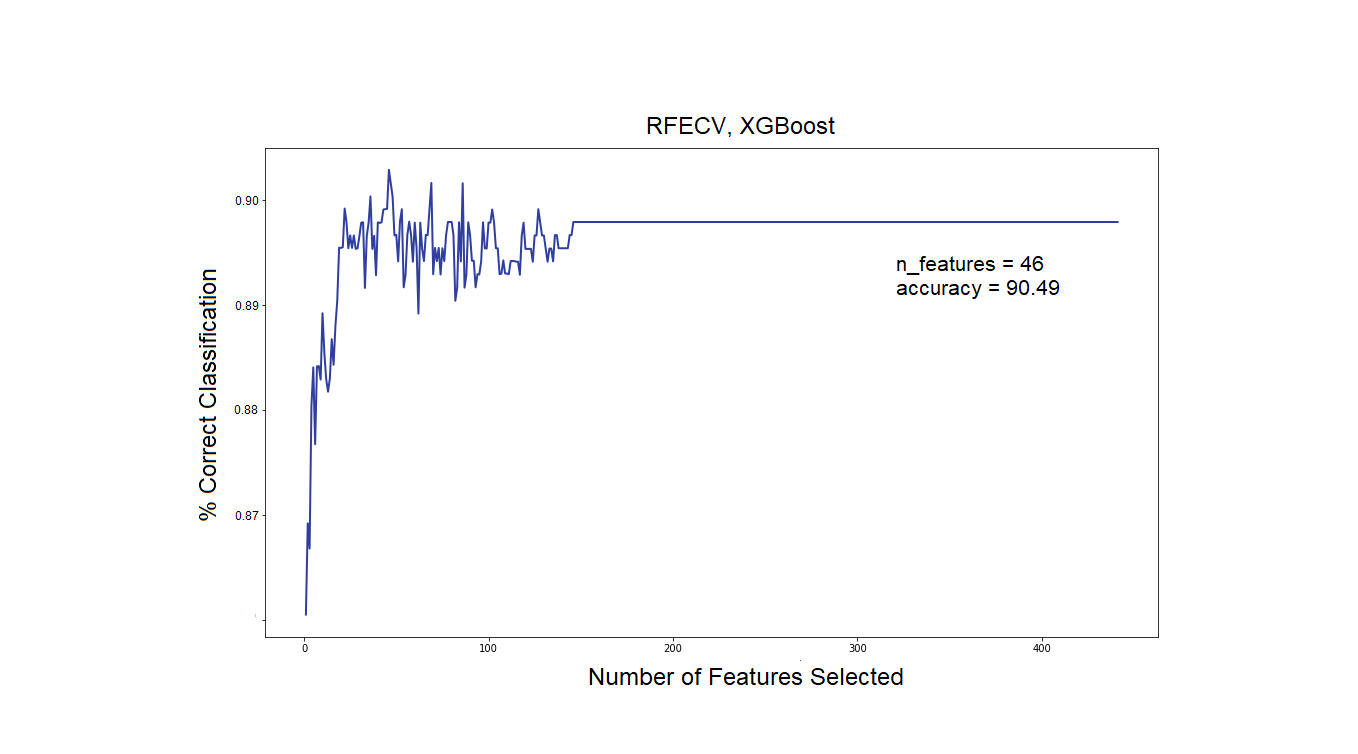

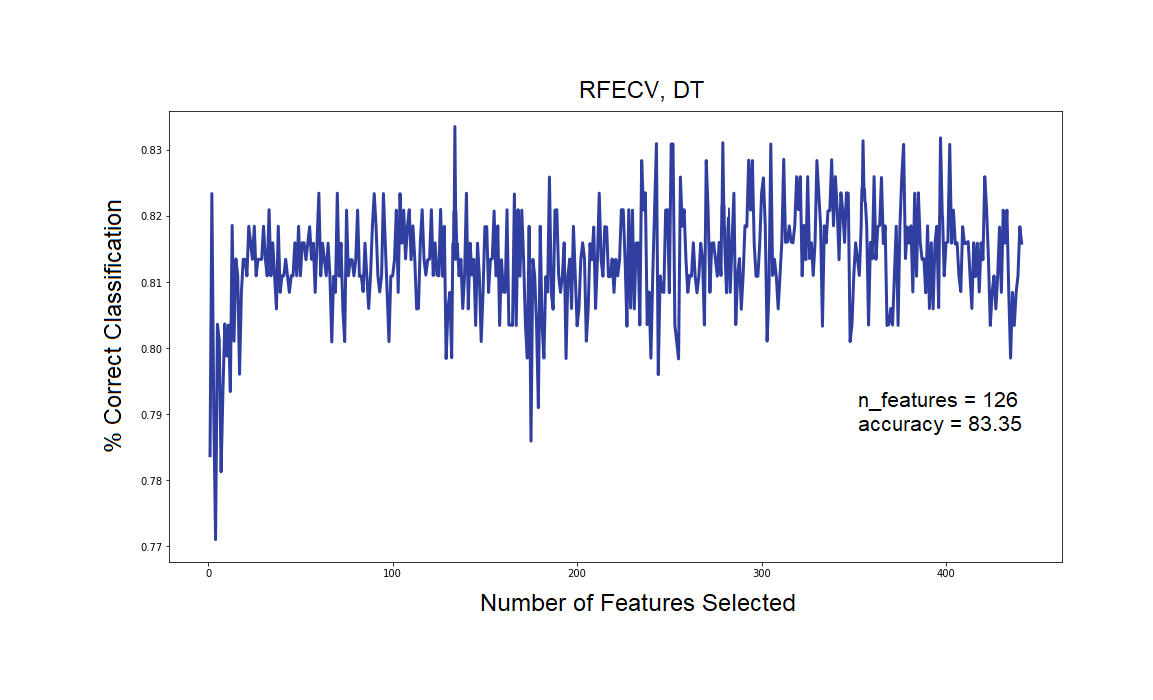


A

B

C

C


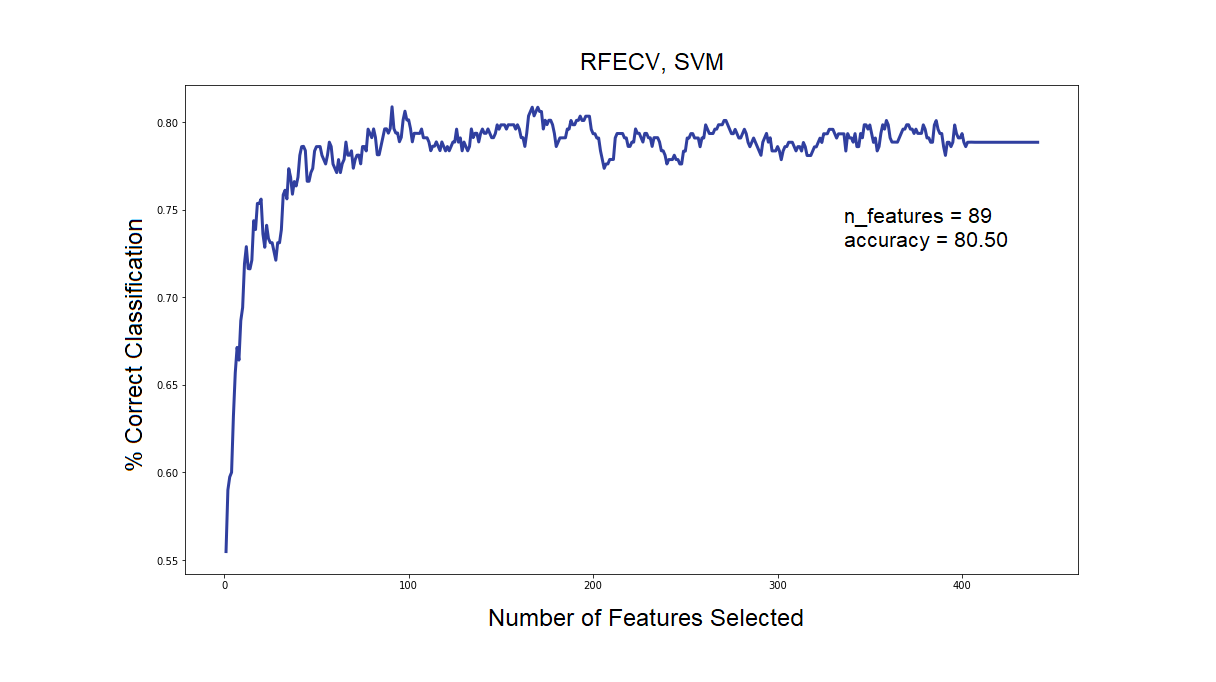

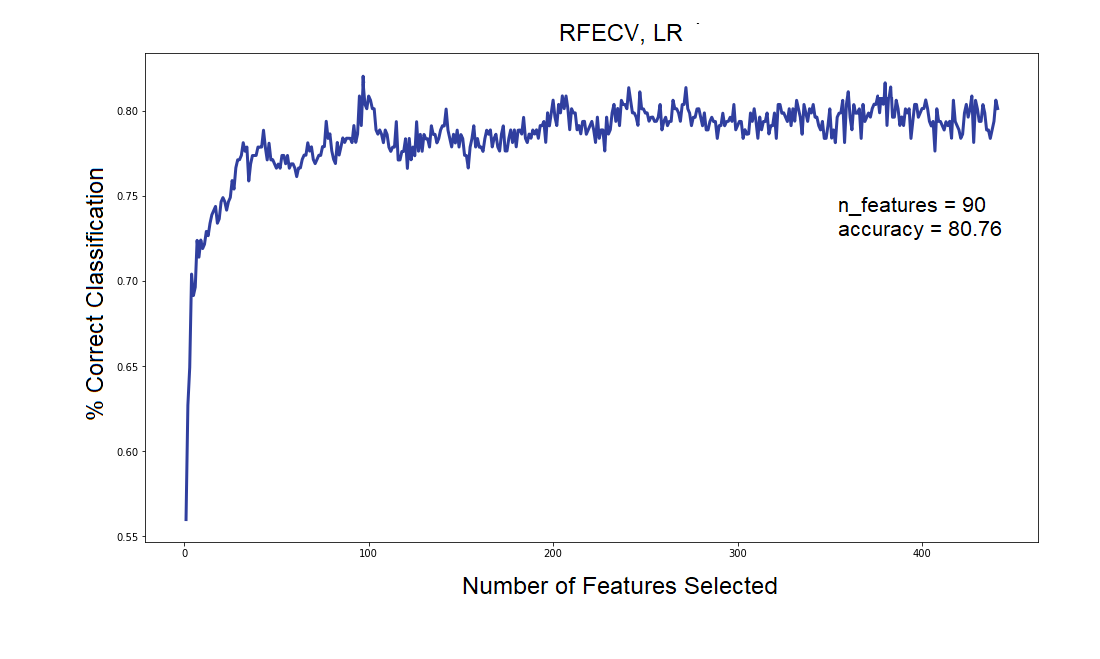


D

EE

**Figure S1: RFECV curves for five machine learning model. As MLP doesn’t have feature importance attribute, XGBoost was used as base estimator. Maximum 128 features have been selected by decision tree whereas, minimum 46 have been captured with XGBoost by RFECV. (A) RFECV curve for XGBoost, (B) RFECV curve for RF, (C) RFECV curve for DT, (D) RFECV curve for SVM, (E) RFECV curve for LR.**

**Figure S2: A scatter plot between inhibitory potency and lipophilicity representing LipE values of training set compounds. 309 training set compounds out of 658 showed LipE value below 1 whereas only 151 could be able to cross the optimal threshold of 5.**

**Figure S3: Graph showing relationship between lipophilicity and inhibitory potency of all six classes of compounds. The value of R^2^ for class I (circle) is 0.57, for class II (triangle) is 0.3, for class III (star) is 0.01, for class IV (plus) is 0.09, for class V (rectangle) is 0.15, and for class VI (rhombus) is 0.032.**

**Table S3: Data set of 151 compounds used for molecular docking and GRIND analysis.**

| **#** | **CMPD_CHEMBLID** | **CANONICAL_SMILES** | **IC_50_** | **PIC_50_** | **clogP** | **LipE** |
| --- | --- | --- | --- | --- | --- | --- |
| 1 | CHEMBL1922663 | S(C(C)(C)C)c1c(CC(C(=O)O)(C)C)n(Cc2ccc(-c3ncc(F)cc3)cc2)c2c1cc(OCc1ncc(C)cc1)cc2 | 0.40 | 9.40 | 8.06 | 1.34 |
| 2 | CHEMBL313489 | Clc1ccc(Cn2c(CC(C(=O)[O-])(C)C)c(SC(C)(C)C)c3c2ccc(OCc2nc4c(cc2)cccc4)c3)cc1 | 0.50 | 9.30 | 4.82 | 4.48 |
| 3 | CHEMBL1922665 | S(C(C)(C)C)c1c(CC(C(=O)O)(C)C)n(Cc2ccc(-c3cnccc3)cc2)c2c1cc(OCc1ncc(C)cc1)cc2 | 0.52 | 9.30 | 7.62 | 1.68 |
| 4 | CHEMBL557527 | S(C(C)(C)C)c1c(CC(C(=O)O)(C)C)n(Cc2ccc(-c3cnc(OC)cc3)cc2)c2c1cc(OCc1ncccc1)cc2 | 0.57 | 9.34 | 7.94 | 1.36 |
| 5 | CHEMBL1229205 | S(C(C)(C)C)c1c(CC(C(=O)O)(C)C)n(Cc2ccc(-c3ncc(OC)cn3)cc2)c2c1cc(OCc1ncc(C)cc1)cc2 | 0.60 | 9.22 | 7.42 | 1.80 |
| 6 | CHEMBL3681322 | CC(C)(C)n1c(nc2cc(ccc12)c3cnc(N)nc3)c4ccccc4C(=O)Nc5ccncc5 | 0.91 | 9.04 | 3.78 | 5.26 |
| 7 | CHEMBL4088763 | O=C(N(C(C)(C)C)C)Cn1ncc(-c2onc(C3(c4ccc(-c5cnc(N)nc5)cc4)CCC3)n2)c1 | 1.00 | 9.00 | 3.13 | 5.87 |
| 8 | CHEMBL4088763 | CN(C(=O)Cn1cc(cn1)c2nc(no2)C3(CCC3)c4ccc(cc4)c5cnc(N)nc5)C(C)(C)C | 1.00 | 9.00 | 3.13 | 5.87 |
| 9 | CHEMBL4088763 | O=C(N(C(C)(C)C)C)Cn1ncc(-c2onc(C3(c4ccc(-c5cnc(N)nc5)cc4)CCC3)n2)c1 | 1.00 | 9.00 | 3.13 | 5.87 |
| 10 | CHEMBL2031647 | S=C1OC(c2c([C@@]3(c4ccccc4)[C@@H]4C[C@@H](C3)CC4)cc(OCc3nc4c(cc3)cccc4)cc2)=NN1 | 1.10 | 8.96 | 7.88 | 1.08 |
| 11 | CHEMBL3684717 | O[C@@H]1CN(c2ncc(-c3onc(C4(c5cnc(-c6cnc(N)nc6)cc5)CCC4)n3)cc2)CC1 | 1.20 | 8.92 | 2.34 | 6.58 |
| 12 | CHEMBL3417526 | CN(C)C(=O)Cn1cc(cn1)c2nc(no2)C(C)(C3CC3)c4ccc(cc4)c5cnc(N)cn5 | 1.20 | 8.92 | 2.35 | 6.57 |
| 13 | CHEMBL3417520 | Nc1ncc(-c2ccc([C@](C)(c3nc(-c4cn(C)nc4)on3)C3CC3)cc2)cn1 | 1.3 | 8.89 | 2.36 | 6.53 |
| 14 | CHEMBL3417520 | Nc1ncc(-c2ccc([C@](C)(c3nc(-c4cn(C)nc4)on3)C3CC3)cc2)cn1 | 1.30 | 8.89 | 2.36 | 6.53 |
| 15 | CHEMBL3417520 | Cn1cc(cn1)c2nc(no2)C(C)(C3CC3)c4ccc(cc4)c5cnc(N)nc5 | 1.30 | 8.89 | 2.36 | 6.53 |
| 16 | CHEMBL3689122 | CC(C)Nc1ccc(cn1)c2nc(no2)C3(CCC3)c4ccc(nc4)c5cnc(N)nc5 | 1.50 | 8.82 | 2.69 | 6.13 |
| 17 | CHEMBL4088331 | Nc1ncc(-c2ncc([C@@](C)(c3nc(-c4cn(C5COC5)nc4)on3)C3CC3)cc2)cn1 | 1.50 | 8.82 | 2.98 | 5.84 |
| 18 | CHEMBL3681336 | CC(C)(C)n1c(nc2cc(ccc12)c3cnc(N)nc3)c4ccccc4C(=O)Nc5cccnc5 | 1.50 | 8.82 | 3.78 | 5.04 |
| 19 | CHEMBL3417518 | Nc1ncc(-c2ccc([C@](C(C)C)(C)c3nc(-c4cn(C)nc4)on3)cc2)cn1 | 1.60 | 8.80 | 3.08 | 5.72 |
| 20 | CHEMBL2031651 | O(Cc1nc2c(cc1)cccc2)c1cc(c(-c2nc(C)on2)cc1)[C@@]1(c2ccccc2)[C@@H]2C[C@@H](C1)CC2 | 1.60 | 8.80 | 7.71 | 1.09 |
| 21 | CHEMBL3417518 | CC(C)C(C)(c1ccc(cc1)c2cnc(N)nc2)c3noc(n3)c4cnn(C)c4 | 1.60 | 8.80 | 3.08 | 5.72 |
| 22 | CHEMBL3417518 | Nc1ncc(-c2ccc([C@](C(C)C)(C)c3nc(-c4cn(C)nc4)on3)cc2)cn1 | 1.60 | 8.80 | 3.08 | 5.72 |
| 23 | CHEMBL38958 | Clc1ccc(Cn2c(CC(C(=O)O)(C)C)c(SC(C)(C)C)c3c2ccc(OCc2ncccc2)c3)cc1 | 1.70 | 8.77 | 7.43 | 1.34 |
| 24 | CHEMBL3417525 | CN(C)C(=O)Cn1cc(cn1)c2nc(no2)C(C)(C3CC3)c4ccc(cc4)c5cnc(N)nc5 | 1.70 | 8.77 | 2.14 | 6.63 |
| 25 | CHEMBL3417525 | O=C(N(C)C)Cn1ncc(-c2onc([C@@](C)(c3ccc(-c4cnc(N)nc4)cc3)C3CC3)n2)c1 | 1.70 | 8.77 | 2.14 | 6.63 |
| 26 | CHEMBL3417429 | Cn1cc(cn1)c2nc(no2)C3(CCC3)c4ccc(cc4)c5cnc(N)nc5 | 1.90 | 8.72 | 2.11 | 6.61 |
| 27 | CHEMBL1210423 | [Na+].COc1cnc(nc1)c5ccc(Cn3c(CC(C)(C)C([O-])=O)c(SC(C)(C)C)c4cc(OCc2ccc(C)cn2)ccc34)cc5 | 2.00 | 8.70 | 3.42 | 5.28 |
| 28 | CHEMBL1210423 | S(C(C)(C)C)c1c(CC(C(=O)[O-])(C)C)n(Cc2ccc(-c3ncc(OC)cn3)cc2)c2c1cc(OCc1ncc(C)cc1)cc2 | 2.00 | 8.70 | 3.42 | 5.28 |
| 29 | CHEMBL1229205 | COc1cnc(nc1)c5ccc(Cn3c(CC(C)(C)C(O)=O)c(SC(C)(C)C)c4cc(OCc2ccc(C)cn2)ccc34)cc5 | 2.00 | 8.70 | 7.42 | 1.28 |
| 30 | CHEMBL3684682 | CCNc1ccc(cn1)c2nc(no2)C3(CCC3)c4ccc(nc4)c5cnc(N)nc5 | 2.00 | 8.70 | 2.38 | 6.32 |
| 31 | CHEMBL3417517 | CC(C)C(C)(c1ccc(cc1)c2cnc(N)nc2)c3noc(n3)c4cnn(C)c4 | 2.00 | 8.70 | 3.08 | 5.62 |
| 32 | CHEMBL2029369 | O=C(OC)c1c([C@@]2(c3ccccc3)[C@@H]3C[C@@H](C2)CC3)cc(OCc2nc3c(cc2)cccc3)cc1 | 2.10 | 8.68 | 2.97 | 5.71 |
| 33 | CHEMBL3681348 | CC(C)(C)n1c(nc2cc(ccc12)c3cnc(N)nc3)c4ccccc4C(=O)Nc5ccccn5 | 2.20 | 8.66 | 3.78 | 4.88 |
| 34 | CHEMBL2031437 | Fc1cc([C@]2(c3c(C(=O)OC)ccc(OCc4nc5c(cc4)cccc5)c3)[C@@H]3C[C@@H](C2)CC3)ccc1 | 2.30 | 8.64 | 2.67 | 5.97 |
| 35 | CHEMBL2031656 | FC(F)n1nnc(n1)c4ccc(OCc3ccc2ccccc2n3)cc4C5(CC6CCC5C6)c7ccccc7 | 2.40 | 8.62 | 7.60 | 1.02 |
| 36 | CHEMBL3417431 | COCCn1cc(cn1)c2nc(no2)C3(CCC3)c4ccc(cc4)c5cnc(N)nc5 | 2.40 | 8.62 | 2.18 | 6.44 |
| 37 | CHEMBL3417427 | Nc1ncc(cn1)c2ccc(cc2)C3(CCC3)c4noc(n4)c5cc[nH]n5 | 2.40 | 8.62 | 2.59 | 6.03 |
| 38 | CHEMBL3417425 | Nc1ncc(cn1)c2ccc(cc2)C3(CCC3)c4noc(n4)c5cscn5 | 2.40 | 8.62 | 2.69 | 5.93 |
| 39 | CHEMBL2031656 | FC(F)n1nc(-c2c([C@@]3(c4ccccc4)[C@@H]4C[C@@H](C3)CC4)cc(OCc3nc4c(cc3)cccc4)cc2)nn1 | 2.40 | 8.62 | 7.60 | 1.02 |
| 40 | CHEMBL558778 | O=C(Oc1c(C2(c3ccccc3)C3CC(C2)CC3)cc(OCc2sc3c(n2)cccc3)cc1)Nc1cnccc1 | 2.40 | 8.62 | 2.38 | 6.24 |
| 41 | CHEMBL610472 | S(C(C)(C)C)c1c(CC(C(=O)O)(C)C)n(Cc2ccc(-c3nc(OC)sc3)cc2)c2c1cc(OC[C@H]1N(C(=O)C)c3c(cccc3)C1)cc2 | 2.50 | 8.60 | 2.18 | 6.42 |
| 42 | CHEMBL610472 | S(C(C)(C)C)c1c(CC(C(=O)O)(C)C)n(Cc2ccc(-c3nc(OC)sc3)cc2)c2c1cc(OC[C@H]1N(C(=O)C)c3c(cccc3)C1)cc2 | 2.50 | 8.60 | 2.18 | 6.42 |
| 43 | CHEMBL3417422 | Nc1ncc(cn1)c2ccc(cc2)C3(CCC3)c4noc(n4)c5c[nH]cn5 | 2.50 | 8.60 | 2.27 | 6.33 |
| 44 | CHEMBL3684736 | Nc1ncc(cn1)c2ccc(cn2)C3(CCC3)c4noc(n4)c5ccc(cc5)N6CCNCC6 | 2.60 | 8.59 | 2.31 | 6.28 |
| 45 | CHEMBL31540 | Clc1ccc(Cn2c(CC(C(=O)O)(C)C)c(C)c3c2ccc(OCc2nc4c(cc2)cccc4)c3)cc1 | 2.80 | 8.55 | 7.24 | 1.31 |
| 46 | CHEMBL2031653 | Cn1nnc(n1)c4ccc(OCc3ccc2ccccc2n3)cc4C5(CC6CCC5C6)c7ccccc7 | 2.80 | 8.55 | 7.41 | 1.14 |
| 47 | CHEMBL2031649 | O(Cc1nc2c(cc1)cccc2)c1cc(c(-c2ocnn2)cc1)[C@@]1(c2ccccc2)[C@@H]2C[C@@H](C1)CC2 | 2.90 | 8.54 | 6.57 | 1.97 |
| 48 | CHEMBL3684706 | Nc1ncc(cn1)c2ccc(cn2)C3(CCC3)c4noc(n4)c5ccc(O)cc5 | 2.90 | 8.54 | 2.51 | 6.03 |
| 49 | CHEMBL3684683 | Nc1ncc(cn1)c2ccc(cn2)C3(CCC3)c4noc(n4)c6ccc(NC5CC5)nc6 | 2.90 | 8.54 | 2.66 | 5.88 |
| 50 | CHEMBL3417527 | CNc1ncc(cn1)c2ccc(cc2)C(C)(C3CC3)c4noc(n4)c5cnn(CC(=O)N(C)C)c5 | 2.90 | 8.54 | 3.20 | 5.34 |
| 51 | CHEMBL1922660 | S(C(C)(C)C)c1c(CC(C(=O)O)(C)C)n(Cc2ccc(-c3cnc(OCC)cc3)cc2)c2c1cc(OCc1ncc(C)cc1)cc2 | 2.90 | 8.54 | 7.41 | 1.13 |
| 52 | CHEMBL2031649 | O(Cc1nc2c(cc1)cccc2)c1cc(c(-c2ocnn2)cc1)[C@@]1(c2ccccc2)[C@@H]2C[C@@H](C1)CC2 | 2.90 | 8.54 | 7.37 | 1.17 |
| 53 | CHEMBL4072041 | O=C(N(C)C)Cn1ncc(-c2onc([C@@](C)(c3cnc(-c4cnc(N)nc4)cc3)C3CC3)n2)c1 | 2.90 | 8.54 | 2.75 | 5.79 |
| 54 | CHEMBL3417527 | O=C(N(C)C)Cn1ncc(-c2onc([C@](C)(c3ccc(-c4cnc(NC)nc4)cc3)C3CC3)n2)c1 | 2.90 | 8.54 | 6.57 | 1.97 |
| 55 | CHEMBL38958 | CC(C)(C)Sc3c(CC(C)(C)C(O)=O)n(Cc1ccc(Cl)cc1)c4ccc(OCc2ccccn2)cc34 | 3.00 | 8.52 | 7.43 | 1.09 |
| 56 | CHEMBL3689125 | COCCN(C)c1ccc(cn1)c2nc(no2)C3(CCC3)c4ccc(nc4)c5cnc(N)nc5 | 3.00 | 8.52 | 2.07 | 6.45 |
| 57 | CHEMBL4080822 | CC(C)C(C)(c1ccc(cc1)c2cnc(N)nc2)c3noc(n3)N4CCC(C)(O)CC4 | 3.10 | 8.51 | 3.07 | 5.44 |
| 58 | CHEMBL3417530 | CN(C)CCn1cc(cn1)c2nc(no2)C(C)(C3CC3)c4ccc(cc4)c5cnc(N)nc5 | 3.10 | 8.51 | 2.62 | 5.89 |
| 59 | CHEMBL594773 | COC1=CC=C(NN1)c6ccc(Cn4c(CC(C)(C)C(O)=O)c(SC(C)(C)C)c5cc(OCC3Cc2ccccc2N3C(C)=O)ccc45)cc6 | 3.20 | 8.49 | 7.23 | 1.26 |
| 60 | CHEMBL3641489 | CC(C)(C)n1c(nc2cc(ccc12)c3cnc(N)nc3)c4cc(Cl)cnc4n5cncn5 | 3.20 | 8.49 | 3.08 | 5.41 |
| 61 | CHEMBL557548 | S(C(C)(C)C)c1c(CC(C(=O)O)(C)C)n(Cc2ccc(-c3nc(OC)sc3)cc2)c2c1cc(OCc1ncccc1)cc2 | 3.30 | 8.48 | 7.23 | 1.25 |
| 62 | CHEMBL3689120 | Nc1ncc(cn1)c2ccc(cn2)C3(CCC3)c4noc(n4)c5ccc(nc5)N6CCCC6 | 3.30 | 8.48 | 2.76 | 5.72 |
| 63 | CHEMBL3417511 | CC(C)(O)Cn1cc(cn1)c2nc(no2)C3(CCC3)c4ccc(cc4)c5cnc(N)nc5 | 3.50 | 8.46 | 2.13 | 6.33 |
| 64 | CHEMBL403199 | O=C(OCC)CCCOc1c(C2(c3ccccc3)C3CC(C2)CC3)cc(OCc2nc3c(cc2)cccc3)cc1 | 3.70 | 8.43 | 2.37 | 6.06 |
| 65 | CHEMBL3689181 | CC(C)(C)CC(=O)N1CCN(CC1)c2ccc(cn2)c3nc(no3)C4(CCC4)c5ccc(nc5)c6cnc(N)nc6 | 3.80 | 8.42 | 3.03 | 5.39 |
| 66 | CHEMBL552567 | COc1ccc(nn1)c5ccc(Cn3c(CC(C)(C)C(O)=O)c(SC(C)(C)C)c4cc(OCc2ccccn2)ccc34)cc5 | 4.00 | 8.40 | 6.79 | 1.61 |
| 67 | CHEMBL552567 | S(C(C)(C)C)c1c(CC(C(=O)O)(C)C)n(Cc2ccc(-c3nnc(OC)cc3)cc2)c2c1cc(OCc1ncccc1)cc2 | 4.00 | 8.40 | 7.39 | 1.01 |
| 68 | CHEMBL3684752 | Nc1ncc(cn1)c2ccc(cn2)C3(CCC3)c4noc(n4)c6ccc5[nH]ncc5c6 | 4.00 | 8.40 | 2.40 | 6.00 |
| 69 | CHEMBL254747 | O(Cc1ncccc1)c1c(C2(c3ccccc3)C3CC(C2)CC3)cc(OCc2nc3c(cc2)cccc3)cc1 | 4.10 | 8.39 | 6.79 | 1.60 |
| 70 | CHEMBL3641469 | N#Cc1nc(-c2n(C(C)(C)C)c3c(n2)cc(-c2cnc(N)nc2)cc3)c(-n2nc(C)nc2)cc1 | 4.20 | 8.38 | 2.54 | 5.84 |
| 71 | CHEMBL610471 | CC(=O)N4C(COc3ccc2n(Cc1ccc(Cl)cc1)c(CC(C)(C)C(O)=O)c(c2c3)S(=O)(=O)C(C)(C)C)Cc5ccccc45 | 4.50 | 8.35 | 6.62 | 1.73 |
| 72 | CHEMBL1922668 | S(C(C)(C)C)c1c(CC(C(=O)O)(C)C)n(Cc2ccc(-c3cnc(OCC)cc3)cc2)c2c1cc(OCc1[n+]([O-])cc(C)cc1)cc2 | 4.60 | 8.34 | 6.62 | 1.72 |
| 73 | CHEMBL610469 | Clc1ccc(Cn2c(CC(C(=O)O)(C)C)c(CCC(C)(C)C)c3c2ccc(OC[C@H]2N(C(=O)C)c4c(cccc4)C2)c3)cc1 | 4.70 | 8.33 | 7.09 | 1.24 |
| 74 | CHEMBL539650 | CC(C)(C)Sc4c(CC(C)(C)C(O)=O)n(Cc1ccc(cc1)c2nccs2)c5ccc(OCc3ccccn3)cc45 | 4.80 | 8.32 | 7.31 | 1.01 |
| 75 | CHEMBL2031648 | Cc1nnc(o1)c4ccc(OCc3ccc2ccccc2n3)cc4C5(CC6CCC5C6)c7ccccc7 | 5.00 | 8.30 | 6.84 | 1.46 |
| 76 | CHEMBL3681324 | CNC(=O)c1ccc(F)cc1c3nc2cc(ccc2n3C(C)(C)C)c4cnc(N)nc4 | 5.07 | 8.29 | 3.00 | 5.29 |
| 77 | CHEMBL4081751 | OC1CCN(c2onc([C@](C)(c3ccc(-c4cnc(N)nc4)cc3)C3CC3)n2)CC1 | 5.20 | 8.28 | 2.74 | 5.54 |
| 78 | CHEMBL4105047 | CC1(O)CCN(CC1)c2nc(no2)C(C)(C3CC3)c4ccc(cc4)c5cnc(N)nc5 | 5.60 | 8.25 | 2.36 | 5.89 |
| 79 | CHEMBL3684680 | Cc1ccc(cc1)c2nc(no2)C3(CCC3)c4ccc(nc4)c5cnc(N)nc5 | 5.80 | 8.24 | 3.19 | 5.05 |
| 80 | CHEMBL2031456 | O=C(Nc1cnccc1)c1c([C@@]2(c3ccccc3)[C@@H]3C[C@@H](C2)CC3)cc(OCc2nc3c(cc2)cccc3)cc1 | 5.80 | 8.24 | 7.04 | 1.20 |
| 81 | CHEMBL3641470 | Cc1ncnn1c2ccc(C#N)nc2c4nc3cc(ccc3n4C(C)(C)C)c5cnc(N)nc5 | 5.90 | 8.23 | 2.54 | 5.69 |
| 82 | CHEMBL3681328 | O=C(NC#N)c1c(-c2n(C(C)(C)C)c3c(n2)cc(-c2cnc(N)nc2)cc3)cc(C#N)cc1 | 6.09 | 8.22 | 2.18 | 6.26 |
| 83 | CHEMBL4074281 | Nc1ncc(-c2ccc(C3(c4nc(N5CCCCC5)on4)CCC3)cc2)cn1 | 6.5 | 8.19 | 3.91 | 4.28 |
| 84 | CHEMBL4098484 | Nc1ncc(-c2ncc([C@](C(C)C)(C)c3nc(-c4cn(C5COC5)nc4)on3)cc2)cn1 | 6.60 | 8.18 | 3.91 | 4.27 |
| 85 | CHEMBL557052 | CC(C)(C)Sc4c(CC(C)(C)C(O)=O)n(Cc1ccc(cc1)c2ncccn2)c5ccc(OCc3ccccn3)cc45 | 6.90 | 8.16 | 6.36 | 1.80 |
| 86 | CHEMBL557052 | S(C(C)(C)C)c1c(CC(C(=O)O)(C)C)n(Cc2ccc(-c3ncccn3)cc2)c2c1cc(OCc1ncccc1)cc2 | 6.90 | 8.16 | 2.47 | 5.69 |
| 87 | CHEMBL78938 | Clc1ccc(Cn2c(CC(C(=O)O)(C)C)c(SC(C)(C)C)c3c2ccc(OC(C)c2nc4c(cc2)cccc4)c3)cc1 | 7.00 | 8.15 | 2.92 | 5.23 |
| 88 | CHEMBL79383 | CC(C)(C)Sc3c(CC(C)(C)C(O)=O)n(Cc1ccc(Cl)cc1)c4ccc(OCc2cnccn2)cc34 | 7.00 | 8.15 | 6.47 | 1.68 |
| 89 | CHEMBL4099770 | CC(C)C(C)(c1ccc(cc1)c2cnc(N)nc2)c3noc(n3)N4CCC(O)CC4 | 7.30 | 8.14 | 2.56 | 5.58 |
| 90 | CHEMBL4099770 | OC1CCN(c2onc([C@@](C(C)C)(C)c3ccc(-c4cnc(N)nc4)cc3)n2)CC1 | 7.30 | 8.14 | 6.47 | 1.67 |
| 91 | CHEMBL3681326 | CNC(=O)c1ccc(C#N)cc1c3nc2cc(ccc2n3C(C)(C)C)c4cnc(N)nc4 | 7.40 | 8.13 | 2.55 | 5.58 |
| 92 | CHEMBL3684743 | Nc1ncc(cn1)c2ccc(cn2)C3(CCC3)c4noc(n4)c5cnn(CC(F)(F)F)c5 | 7.60 | 8.12 | 2.53 | 5.59 |
| 93 | CHEMBL555207 | CC(C)(C)Sc4c(CC(C)(C)C(O)=O)n(Cc1ccc(cc1)c2cncnc2)c5ccc(OCc3ccccn3)cc45 | 7.70 | 8.11 | 6.15 | 1.96 |
| 94 | CHEMBL3417519 | CC(C)C(C)(c1ccc(cc1)c2cnc(N)nc2)c3noc(n3)c4cnn(C)c4 | 7.70 | 8.11 | 3.08 | 5.03 |
| 95 | CHEMBL1922667 | Cc5ccc(COc4ccc3n(Cc1ccc(cc1)c2ccc(=O)[nH]c2)c(CC(C)(C)C(O)=O)c(SC(C)(C)C)c3c4)nc5 | 8.30 | 8.08 | 6.40 | 1.68 |
| 96 | CHEMBL4101030 | Nc1ncc(cn1)c2ccc(cc2)C3(CCC3)c4noc(n4)N5CCCC5 | 8.30 | 8.08 | 3.35 | 4.73 |
| 97 | CHEMBL3684679 | Cc1cccc(c1)c2nc(no2)C3(CCC3)c4ccc(nc4)c5cnc(N)nc5 | 8.80 | 8.06 | 3.19 | 4.87 |
| 98 | CHEMBL538632 | S(C(C)(C)C)c1c(CC(C(=O)O)(C)C)n(Cc2ccc(-c3cnccc3)cc2)c2c1cc(OCc1ncccc1)cc2 | 9.00 | 8.05 | 7.11 | 0.94 |
| 99 | CHEMBL3684701 | Nc1ncc(cn1)c2ccc(cn2)C3(CCC3)c4noc(n4)c6ccc5nccn5c6 | 12.40 | 7.91 | 2.19 | 5.72 |
| 100 | CHEMBL4087927 | CC1(O)CN(C1)c2nc(no2)C(C)(C3CC3)c4ccc(cc4)c5cnc(N)nc5 | 16.00 | 7.80 | 2.59 | 5.21 |
| 101 | CHEMBL4078361 | CC1(O)CCN(CC1)c2nc(no2)C3(CCC3)c4ccc(cc4)c5cnc(N)nc5 | 17.00 | 7.77 | 2.11 | 5.66 |
| 102 | CHEMBL3641494 | Cc1ccc(c(n1)n2cncn2)c4nc3cc(ccc3n4C(C)(C)C)c5cnc(N)nc5 | 17.00 | 7.77 | 2.87 | 4.90 |
| 103 | CHEMBL3681337 | CCNC(=O)c1ccccc1c3nc2cc(ccc2n3C(C)(C)C)c4cnc(N)nc4 | 17.40 | 7.76 | 3.27 | 4.49 |
| 104 | CHEMBL3417521 | Cn1cc(cn1)c2nc(no2)C(C)(C3CC3)c4ccc(cc4)c5cnc(N)nc5 | 18.00 | 7.74 | 2.36 | 5.38 |
| 105 | CHEMBL3681335 | CC(C)(C)n1c(nc2cc(ccc12)c3cnc(N)nc3)c4ccccc4C(=O)NCC5CCCO5 | 18.80 | 7.73 | 3.38 | 4.35 |
| 106 | CHEMBL3641488 | CC(C)(C)n1c(nc2cc(ccc12)c3cnc(N)nc3)c4cc(F)cnc4n5cncn5 | 20.00 | 7.70 | 2.51 | 5.19 |
| 107 | CHEMBL3681344 | CNC(=O)c1cc(F)ccc1c3nc2cc(ccc2n3C(C)(C)C)c4cnc(N)nc4 | 20.00 | 7.70 | 3.00 | 4.70 |
| 108 | CHEMBL4084576 | CN(C)c1nc(no1)C2(CCC2)c3ccc(cc3)c4cnc(N)nc4 | 21.00 | 7.68 | 2.65 | 5.03 |
| 109 | CHEMBL3681325 | CNC(=O)c1cc(OC)ccc1c3nc2cc(ccc2n3C(C)(C)C)c4cnc(N)nc4 | 21.90 | 7.66 | 2.89 | 4.77 |
| 110 | CHEMBL4068746 | N#CCn1ncc(-c2onc([C@@](C(C)C)(C)c3cnc(-c4cnc(N)nc4)cc3)n2)c1 | 23.00 | 7.64 | 1.43 | 6.21 |
| 111 | CHEMBL3641491 | Cc1ncnn1c2ncccc2c4nc3cc(ccc3n4C(C)(C)C)c5cnc(N)nc5 | 23.00 | 7.64 | 2.64 | 5.00 |
| 112 | CHEMBL3681321 | CC(C)(C)n1c(nc2cc(ccc12)c3cnc(N)nc3)c4ccccc4C(=O)NC5CC5 | 25.20 | 7.60 | 3.33 | 4.27 |
| 113 | CHEMBL541967 | CC(C)(C)Sc4c(CC(C)(C)C(O)=O)n(Cc1ccc(cc1)c2cnccn2)c5ccc(OCc3ccccn3)cc45 | 28.00 | 7.55 | 6.36 | 1.19 |
| 114 | CHEMBL4095628 | N(C)(c1onc([C@@](C)(c2ccc(-c3cnc(N)nc3)cc2)C2CC2)n1)c1cn(C)nc1 | 29.00 | 7.54 | 2.79 | 4.75 |
| 115 | CHEMBL4095628 | N(C)(c1onc([C@@](C)(c2ccc(-c3cnc(N)nc3)cc2)C2CC2)n1)c1cn(C)nc1 | 29.00 | 7.54 | 4.86 | 2.68 |
| 116 | CHEMBL3689180 | CNc1cnc(cn1)c2ccc(cn2)C3(CCC3)c4noc(n4)c5cnn(CC(C)(C)O)c5 | 32.00 | 7.49 | 2.24 | 5.25 |
| 117 | CHEMBL3689174 | CNc1cnc(cn1)c2ccc(cn2)C3(CCC3)c4noc(n4)c5cnn(C)c5 | 33.00 | 7.48 | 2.22 | 5.26 |
| 118 | CHEMBL3689134 | CN(C)c1ncccc1c2nc(no2)C3(CCC3)c4ccc(nc4)c5cnc(N)nc5 | 34.00 | 7.47 | 2.05 | 5.42 |
| 119 | CHEMBL3641499 | CCOc1ncccc1c3nc2cc(ccc2n3C(C)(C)C)c4cnc(N)nc4 | 34.00 | 7.47 | 3.72 | 3.75 |
| 120 | CHEMBL3681340 | CC(C)NC(=O)c1ccccc1c3nc2cc(ccc2n3C(C)(C)C)c4cnc(N)nc4 | 34.60 | 7.46 | 3.58 | 3.88 |
| 121 | CHEMBL607035 | CC(C)(C)Sc2c(CC(C)(C)C(O)=O)n(Cc1ccc(Cl)cc1)c3ccc(OCC(N)=O)cc23 | 38.00 | 7.42 | 5.66 | 1.76 |
| 122 | CHEMBL3681347 | COCCNC(=O)c1ccccc1c3nc2cc(ccc2n3C(C)(C)C)c4cnc(N)nc4 | 43.80 | 7.36 | 2.94 | 4.42 |
| 123 | CHEMBL4070163 | Nc1ncc(cn1)c2ccc(cc2)C3(CCC3)c4noc(n4)N5CCC(CO)CC5 | 46.00 | 7.34 | 2.44 | 4.90 |
| 124 | CHEMBL4097345 | CC(C)OC1CCN(CC1)c2nc(no2)C3(CCC3)c4ccc(cc4)c5cnc(N)nc5 | 48.00 | 7.32 | 3.01 | 4.31 |
| 125 | CHEMBL4060091 | O=C(N(C)C)Cn1ncc(-c2onc([C@@](C(C)C)(C)c3cnc(-c4cnc(N)nc4)cc3)n2)c1 | 54.00 | 7.27 | 3.01 | 4.26 |
| 126 | CHEMBL3681329 | CC(C)(C)n1c(nc2cc(ccc12)c3cnc(N)nc3)c4cc(C#N)ccc4C(=O)NS(C)(=O)=O | 55.60 | 7.25 | 2.18 | 5.07 |
| 127 | CHEMBL3641484 | COc1cc(c(F)cn1)c3nc2cc(ccc2n3C(C)(C)C)c4cnc(N)nc4 | 58.00 | 7.24 | 3.90 | 3.34 |
| 128 | CHEMBL4094923 | Cc1cc([nH]n1)c2nc(no2)C3(CCC3)c4ccc(cc4)c5cnc(N)nc5 | 80.00 | 7.10 | 2.63 | 4.47 |
| 129 | CHEMBL3684750 | Nc1ncc(cn1)c2ccc(cn2)C3(CCC3)c4noc(n4)N5CCCC5 | 92.00 | 7.04 | 2.20 | 4.84 |
| 130 | CHEMBL3641476 | COC(=O)c1ccc(C#N)nc1c3nc2cc(ccc2n3C(C)(C)C)c4cnc(N)nc4 | 110.00 | 6.96 | 2.94 | 4.02 |
| 131 | CHEMBL3681327 | CNC(=O)c1ccc(cc1c3nc2cc(ccc2n3C(C)(C)C)c4cnc(N)nc4)c5nn[nH]n5 | 110.90 | 6.96 | 2.25 | 4.71 |
| 132 | CHEMBL3681338 | CN(C)CCNC(=O)c1ccccc1c3nc2cc(ccc2n3C(C)(C)C)c4cnc(N)nc4 | 140.00 | 6.85 | 3.15 | 3.70 |
| 133 | CHEMBL3681341 | CN(C(=O)c1ccccc1c3nc2cc(ccc2n3C(C)(C)C)c4cnc(N)nc4)c5ccccn5 | 180.00 | 6.74 | 3.14 | 3.60 |
| 134 | CHEMBL3641496 | CC(C)(C)n1c(nc2cc(ccc12)c3cnc(N)nc3)c4ccc(nc4)N5CCOCC5 | 180.00 | 6.74 | 3.22 | 3.52 |
| 135 | CHEMBL4458304 | Cc1nn(C)cc1NC(=O)C3C2CCC(CC2)C3C(=O)c4ccc(cc4F)c5cc[nH]n5 | 199.53 | 6.70 | 3.17 | 3.53 |
| 136 | CHEMBL3641486 | CC(C)(C)n1c(nc2cc(ccc12)c3cnc(N)nc3)c4ccnc(Br)c4 | 270.00 | 6.57 | 3.84 | 2.73 |
| 137 | CHEMBL552280 | CC(C)(Cc3cc2cc(OCc1ccccn1)ccc2n3Cc4ccc(cc4)c5nccs5)C(O)=O | 299.00 | 6.52 | 5.51 | 1.01 |
| 138 | CHEMBL3417528 | CCNc1ncc(cn1)c2ccc(cc2)C(C)(C3CC3)c4noc(n4)c5cnn(CC(=O)N(C)C)c5 | 370.00 | 6.43 | 3.72 | 2.71 |
| 139 | CHEMBL4239812 | Nc1ncc(cn1)c5ccc(CCc3nc2cc(C#N)ccc2n3Cc4ccccc4Cl)cc5 | 370.00 | 6.43 | 4.98 | 1.45 |
| 140 | CHEMBL258213 | OCCOc1ccc(cc1)C2(CC3CCC2C3)c4ccc(OCCO)cc4 | 387.00 | 6.41 | 4.69 | 1.72 |
| 141 | CHEMBL3641485 | CC(C)(C)n1c(nc2cc(ccc12)c3cnc(N)nc3)c4ccncc4 | 450.00 | 6.35 | 2.93 | 3.42 |
| 142 | CHEMBL3417412 | CCNc1nc(no1)C2(CCC2)c3ccc(cc3)c4cnc(N)nc4 | 460.00 | 6.34 | 3.08 | 3.26 |
| 143 | CHEMBL3681339 | CC(C)(C)n1c(nc2cc(ccc12)c3cnc(N)nc3)c4ccccc4C(=O)NC5CCNCC5 | 460.00 | 6.34 | 2.35 | 3.99 |
| 144 | CHEMBL3681342 | CC(C)(C)n1c(nc2cc(ccc12)c3cnc(N)nc3)c4ccccc4C(=O)N5CCOCC5 | 520.00 | 6.28 | 2.83 | 3.45 |
| 145 | CHEMBL3641474 | COC(=O)c1cccnc1c3nc2cc(ccc2n3C(C)(C)C)c4cnc(N)nc4 | 540.00 | 6.27 | 3.23 | 3.04 |
| 146 | CHEMBL4243603 | Nc1ccc(nn1)c5ccc(CCc3nc2cc(C#N)ccc2n3Cc4ccccc4Cl)cc5 | 660.00 | 6.18 | 5.06 | 1.12 |
| 147 | CHEMBL3641482 | CC(C)(C)n1c(nc2cc(ccc12)c4cnc(NCc3cccc(F)n3)nc4)c5cccc(F)n5 | 870.00 | 6.06 | 4.33 | 1.73 |
| 148 | CHEMBL3641483 | COc1cccnc1c3nc2cc(ccc2n3C(C)(C)C)c4cnc(N)nc4 | 960.00 | 6.02 | 3.00 | 3.02 |
| 149 | CHEMBL3417529 | CC(C)Nc1ncc(cn1)c2ccc(cc2)C(C)(C3CC3)c4noc(n4)c5cnn(CC(=O)N(C)C)c5 | 1,000.00 | 6.00 | 4.03 | 1.97 |
| 150 | CHEMBL3681320 | CC(C)(C)n1c(nc2cc(ccc12)c3cnc(N)nc3)c4ccccc4C(O)=O | 1,400.00 | 5.85 | 3.35 | 2.50 |
| 151 | CHEMBL1289558 | CCC(O)(c4cn(Cc3ccc2c(c1ccc(F)cc1)c(sc2c3)C(N)=O)nn4)C(F)(F)F | 20,000.00 | 4.70 | 3.30 | 1.40 |

**Table S4: Test Set used for validation of GRIND model**

| **#** | **CMPD_CHEMBLID** | **CANONICAL_SMILES** | **IC50** | **PIC50** | **clogP** | **LipE** |
| --- | --- | --- | --- | --- | --- | --- |
| 1 | CHEMBL552545 | CC(C)(C)Sc4c(CC(C)(C)C(O)=O)n(Cc1ccc(cc1)c2ncc(F)s2)c5ccc(OCc3ccccn3)cc45 | 2.1 | 8.68 | 7.50 | 1.18 |
| 2 | CHEMBL3417531 | CC(C)(O)Cn1cc(cn1)c2nc(no2)C(C)(C3CC3)c4ccc(cc4)c5cnc(N)nc5 | 2.1 | 8.68 | 2.38 | 6.30 |
| 3 | CHEMBL3417428 | Nc1ncc(cn1)c2ccc(cc2)C3(CCC3)c4noc(n4)c5cn[nH]c5 | 2.3 | 8.64 | 2.38 | 6.26 |
| 4 | CHEMBL3689118 | CN(C)c1ccc(cn1)c2nc(no2)C3(CCC3)c4ccc(nc4)c5cnc(N)nc5 | 2.4 | 8.62 | 2.05 | 6.57 |
| 5 | CHEMBL3417522 | Cn1cc(cn1)c2nc(no2)C(C)(C3CCC3)c4ccc(cc4)c5cnc(N)nc5 | 2.6 | 8.59 | 2.92 | 5.67 |
| 6 | CHEMBL31540 | Cc4c(CC(C)(C)C(O)=O)n(Cc1ccc(Cl)cc1)c5ccc(OCc3ccc2ccccc2n3)cc45 | 2.8 | 8.55 | 7.46 | 1.09 |
| 7 | CHEMBL3684721 | CCOC(=O)CCN1CCN(CC1)c2ccc(cn2)c3nc(no3)C4(CCC4)c5ccc(nc5)c6cnc(N)nc6 | 3.4 | 8.47 | 3.01 | 5.46 |
| 8 | CHEMBL3417509 | CN(C)CCn1cc(cn1)c2nc(no2)C3(CCC3)c4ccc(cc4)c5cnc(N)nc5 | 3.6 | 8.44 | 2.37 | 6.07 |
| 9 | CHEMBL3641490 | CC(C)(C)n1c(nc2cc(ccc12)c3cnc(N)nc3)c4cc(Br)cnc4n5cncn5 | 4 | 8.40 | 3.23 | 5.17 |
| 10 | CHEMBL605917 | CCC(=O)N1CCCC1COc4ccc3n(Cc2ccc(Cl)cc2)c(CC(C)(C)C(O)=O)c(SC(C)(C)C)c3c4 | 4 | 8.40 | 7.39 | 1.01 |
| 11 | CHEMBL4103286 | CC(C)(CO)n1cc(cn1)c2nc(no2)C3(CCC3)c4ccc(cc4)c5cnc(N)nc5 | 4 | 8.40 | 2.13 | 6.27 |
| 12 | CHEMBL254547 | CS(=O)(=O)CCOc3ccc(OCc2ccc1ccccc1n2)cc3C4(CC5CCC4C5)c6ccccc6 | 5.5 | 8.26 | 7.04 | 1.22 |
| 13 | CHEMBL3684710 | COC(=O)C1CCCN1c2ccc(cn2)c3nc(no3)C4(CCC4)c5ccc(nc5)c6cnc(N)nc6 | 5.9 | 8.23 | 2.26 | 5.97 |
| 13 | CHEMBL3417516 | CC(C)C(c1ccc(cc1)c2cnc(N)nc2)c3noc(n3)c4cnn(C)c4 | 6.2 | 8.21 | 2.68 | 5.53 |
| 15 | CHEMBL4074281 | Nc1ncc(cn1)c2ccc(cc2)C3(CCC3)c4noc(n4)N5CCCCC5 | 6.5 | 8.19 | 3.91 | 4.28 |
| 16 | CHEMBL3689097 | Nc1ncc(cn1)c2ccc(cn2)C3(CCC3)c4noc(n4)c5cnccc5Cl | 7.3 | 8.14 | 2.02 | 6.12 |
| 17 | CHEMBL3689108 | Nc1ncc(cn1)c2ccc(cn2)C3(CCC3)c4noc(n4)c5ccc(Cl)nc5 | 7.5 | 8.12 | 2.27 | 5.85 |
| 18 | CHEMBL3684694 | Cn5cc(c1nc(no1)C2(CCC2)c3ccc(nc3)c4cnc(N)nc4)c(n5)C(F)(F)F | 9.2 | 8.04 | 2.02 | 6.02 |
| 19 | CHEMBL3681323 | CNC(=O)c1ccccc1c3nc2cc(ccc2n3C(C)(C)C)c4cnc(N)nc4 | 11.4 | 7.94 | 2.74 | 5.20 |
| 20 | CHEMBL4059620 | CN(C)C(=O)N1CCN(CC1)c2nc(no2)C(C)(C3CC3)c4ccc(cc4)c5cnc(N)nc5 | 12 | 7.92 | 3.11 | 4.81 |
| 21 | CHEMBL3641471 | CC(C)(C)n1c(nc2cc(ccc12)c3cnc(N)nc3)c4nc(ccc4n5cncn5)C6CC6 | 13 | 7.89 | 3.53 | 4.36 |
| 22 | CHEMBL3689117 | Nc1ncc(cn1)c2ccc(cn2)C3(CCC3)c4noc(n4)c5cccnc5C(F)(F)F | 14 | 7.85 | 2.50 | 5.35 |
| 23 | CHEMBL4568594 | Cc1cc([nH]n1)c2ccc(cc2)C(=O)C3CCCCC3C(=O)Nc4cnn(C)c4C(O)=O | 17 | 7.77 | 3.72 | 4.05 |
| 24 | CHEMBL3681334 | COC(=O)c1ccccc1c3nc2cc(ccc2n3C(C)(C)C)c4cnc(N)nc4 | 22 | 7.66 | 4.32 | 3.34 |
| 25 | CHEMBL3417515 | Cn1cc(cn1)c2nc(no2)C3(CC(C)(C)C3)c4ccc(cc4)c5cnc(N)nc5 | 35 | 7.46 | 3.15 | 4.31 |
| 26 | CHEMBL3684709 | Nc1ncc(cn1)c2ccc(cn2)C3(CCC3)c4noc(n4)c5ccc(nc5)N6CCCC6C(O)=O | 43 | 7.37 | 2.11 | 5.26 |
| 27 | CHEMBL3641480 | CC(C)(C)Oc1cccc(n1)c3nc2cc(ccc2n3C(C)(C)C)c4cnc(N)nc4 | 51 | 7.29 | 5.19 | 2.10 |
| 28 | CHEMBL3689171 | CNc1ncc(cn1)c2ccc(cn2)C3(CCC3)c4noc(n4)c5cnn(CC(C)(C)O)c5 | 73 | 7.14 | 2.03 | 5.11 |
| 29 | CHEMBL4065666 | Nc1ncc(cn1)c2ccc(cc2)C3(CCC3)c4noc(n4)N5CCN(CC5)CC6CCCO6 | 92 | 7.04 | 3.30 | 3.74 |
| 30 | CHEMBL314360 | CC(=O)N(O)CC=Cc2cccc(Oc1ccccc1)c2 | 110 | 6.96 | 3.80 | 3.16 |
| 31 | CHEMBL3641498 | COc1ncccc1c3nc2cc(ccc2n3C(C)(C)C)c4cnc(N)nc4 | 220 | 6.66 | 3.19 | 3.47 |
| 32 | CHEMBL3417415 | Nc1ncc(cn1)c2ccc(cc2)C3(CCC3)c4noc(n4)c5cccnc5 | 250 | 6.60 | 2.67 | 3.93 |
| 33 | CHEMBL4093301 | Nc1ncc(cn1)c2ccc(cc2)C3(CCC3)c4noc(n4)N5CCNCC5 | 324 | 6.49 | 2.41 | 4.08 |
| 34 | CHEMBL3641497 | CC(C)(C)n1c(nc2cc(ccc12)c3cnc(N)nc3)c4cccnc4 | 542 | 6.27 | 2.93 | 3.34 |
| 35 | CHEMBL3641479 | CC(C)(C)n1c(nc2cc(ccc12)c3cnc(N)nc3)c4ccccn4 | 700 | 6.15 | 3.14 | 3.01 |
| 36 | CHEMBL4526540 | Cc1nn(C)cc1NC(=O)C2CCCCC2C(=O)c3ccc(cc3)c4ccc[nH]4 | 1000 | 6.00 | 3.05 | 2.95 |

**Table S5: Details of common scaffold clustering and distribution of compounds in each class.**

| Sr. | Class Name | Common Scaffold | | No. of  Compounds | | Clustered  Compounds | | RMSD  Å | | Total Clusters | | Selected  Cluster | |
| --- | --- | --- | --- | --- | --- | --- | --- | --- | --- | --- | --- | --- | --- |
| **Class I** | Indoles |  | | 32 | | 27 | | 3.5 | | 15 | | 2 [1] | |
| **Class II** | Biaryl bicycloheptane |  | | 20 | | 12 | | 3.5 | | 22 | | 7 [2] | |
| **Class III** | Cyclobutylbe nzene oxadiazole | |  | | 35 | | 32 | | 3.5 | | 11 | | 6 [3] |
| **Class IV** | Cyclopropyle thylbenzene oxadiazole | |  | | 10 | | 10 | | 3.5 | | 8 | | 6 [4] |
| **Class V** | Dimethylpro pylbenzene oxadiazole | |  | | 18 | | 13 | | 3.5 | | 14 | | 5 [5] |
| **Class VI** | Benzimidazoles | |  | | 36 | | 26 | | 3.5 | | 20 | | 12 [6] |

**Table S6: Structural details of R1 and R2 substitutions of compounds displaying distinct SAR pattern from all six classes of FLAP inhibitors along with activity, lipophilicity, and lipophilic efficiency values.**

| **Cmpd.#** | **Class Scaffold** | **R_1_** | **R_2_** | **IC_50_ (nM)** | **-log(IC_50_)** | **cLogP** | **LipE** |
| --- | --- | --- | --- | --- | --- | --- | --- |
| **1** | **I** | 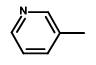 | 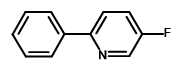 | 0.40 | 9.40 | 8.06 | 1.34 |
| **98** | **I** | 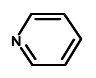 | 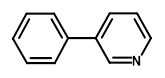 | 9.00 | 8.04 | 3.35 | 4.69 |
| **10** | III |  |  | 1.10 | 8.95 | 7.88 | 1.07 |
| **47** | III |  | 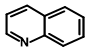 | 2.90 | 8.53 | 7.37 | 1.16 |
| **7** | IV |  |  | 1.00 | 9.00 | 3.13 | 5.87 |
| **83** | IV |  |  | 6.50 | 8.18 | 2.68 | 5.50 |
| **13** | V |  | 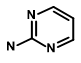 | 1.30 | 8.88 | 2.36 | 6.52 |
| **114** | V |  | 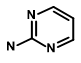 | 29.0 | 7.53 | 4.86 | 2.67 |
| **19** | VI |  | 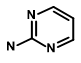 | 1.60 | 8.79 | 3.08 | 5.71 |
| **110** | VI |  | 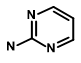 | 23.00 | 7.63 | 2.65 | 4.98 |
| **70** | VII |  | 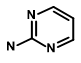 | 4.20 | 8.38 | 2.54 | 5.84 |
| **82** | VII |  | 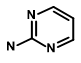 | 6.09 | 8.22 | 2.18 | 6.04 |
